# Phase 1 study to determine the safety and dosing of autologous PBMCs modified to present HPV16 antigens (SQZ-PBMC-HPV) in HLA-A*02+ patients with HPV16+ solid tumors

**DOI:** 10.1007/s10637-023-01342-x

**Published:** 2023-03-03

**Authors:** Antonio Jimeno, Joaquina Baranda, Wade T. Iams, Jong Chul Park, Monica Mita, Michael S. Gordon, Matthew Taylor, Neesha Dhani, Alexis D. Leal, Prakash Neupane, Cathy Eng, Oladapo Yeku, Alain Mita, Justin C. Moser, Marcus Butler, Scott M. Loughhead, Julia Jennings, Nathan R. Miselis, Rui-Ru Ji, Nitya Nair, Martin Kornacker, Ricardo F. Zwirtes, Howard Bernstein, Armon Sharei

**Affiliations:** 1 University of Colorado Comprehensive Cancer Center, 12801 East 17th Avenue, Room L18-8101B, Aurora, CO 80045, USA; 2 University of Kansas Cancer Center, Fairway, KS, USA; 3 Division of Hematology/Oncology, Department of Medicine, Vanderbilt-Ingram Cancer Center, Nashville, TN, USA; 4 Massachusetts General Hospital, Boston, MA, USA; 5 Cedars-Sinai Medical Center, Los Angeles, CA, USA; 6 Pinnacle Oncology Hematology, Arizona Center for Cancer Care, HonorHealth Research Institute Clinical Trials Program, Virginia G. Piper Cancer Center, Scottsdale, AZ, USA; 7 Providence Cancer Institute, Portland, OR, USA; 8 University Health Network Princess Margaret Cancer Centre, Toronto, Canada; 9 SQZ Biotechnologies, Watertown, MA, USA; 10 Hoffmann-La Roche, Basel, Switzerland

**Keywords:** Cellular therapy, HPV16, Antigen presenting cell, Immunotherapy, Cancer vaccine

## Abstract

We conducted a dose escalation Phase 1 study of autologous PBMCs loaded by microfluidic squeezing (Cell Squeeze^®^ technology) with HPV16 E6 and E7 antigens (SQZ-PBMC-HPV), in HLA-A*02+ patients with advanced/metastatic HPV16+ cancers. Preclinical studies in murine models had shown such cells resulted in stimulation and proliferation of antigen specific CD8+ cells, and demonstrated antitumor activity. Administration of SQZ-PBMC-HPV was every 3 weeks. Enrollment followed a modified 3+3 design with primary objectives to define safety, tolerability, and the recommended Phase 2 dose. Secondary and exploratory objectives were antitumor activity, manufacturing feasibility, and pharmacodynamic evaluation of immune responses. Eighteen patients were enrolled at doses ranging from 0.5 × 1 0^6^ to 5.0 × 1 0^6^ live cells/kg. Manufacture proved feasible and required < 24 h within the overall vein-to-vein time of 1 – 2 weeks; at the highest dose, a median of 4 doses were administered. No DLTs were observed. Most related TEAEs were Grade 1 – 2, and one Grade 2 cytokine release syndrome SAE was reported. Tumor biopsies in three patients showed 2 to 8-fold increases in CD8+ tissue infiltrating lymphocytes, including a case that exhibited increased MHC-I+ and PD-L1+ cell densities and reduced numbers of HPV+ cells. Clinical benefit was documented for the latter case. SQZ-PBMC-HPV was well tolerated; 5.0 × 10^6^ live cells/kg with double priming was chosen as the recommended Phase 2 dose. Multiple participants exhibited pharmacodynamic changes consistent with immune responses supporting the proposed mechanism of action for SQZ-PBMC-HPV, including patients previously refractory to checkpoint inhibitors.

## Introduction

The human papilloma virus (HPV) is the most common sexually transmitted infection in the United States and is estimated to cause over 44,000 cases of anal, cervical, head and neck cancers (HNCs), penile, vaginal, and vulvar cancer yearly [1]. The two high-risk HPV subtypes responsible for the majority of HPV-associated cancers are HPV16 and HPV18. For HPV16+ tumors, the epitopes recognized by T cell receptors (TCRs) presented in the context of the HLA-A*02 MHC-I complex have been characterized for the viral proteins E6 and E7 [2–4].

Microfluidic squeezing (Cell Squeeze^®^ technology) was developed as a means of delivering materials diverse in size and type to a variety of cells with minimal impact on cell viability and transcriptional programming [5–7]. Preclinical studies demonstrated this process can be used to cytosolically load antigen-precursors to a variety of immune cells; the cargo-loaded cells process the antigens for MHC-I presentation and can in vitro stimulate antigen-specific CD8+ cells resulting in activation and proliferation of the latter [7, 8]. This circumvents inefficient cross-presentation on professional antigen presenting cells (APCs); in vitro experiments proved it equally effective in both murine and human cells.

Using the murine TC-1 tumor model that expresses HPV16 proteins E6 and E7 [9], immune cells were loaded with a synthetic long peptide (SLP) containing an epitope for E7 and loaded cells were administered 2 weeks after tumor implantation [8]. Significant reduction in tumor growth occurred with associated increases in survival time. Treated tumors showed significant increases in tumor-infiltrating lymphocytes (TILs), with ~30% of the TILs being E7-specific CD8+ T cells compared to 0.2% in untreated mice.

The investigational product SQZ-PBMC-HPV consists of autologous PBMCs presenting immunogenic epitopes of E6 and E7 on HLA-A*02, with the cell mixture consisting primarily of T cells, B cells, natural killer cells, and monocytes. HLA-A*02 is one of the most common MHC-I haplotypes in the United States, ranging from 20 – 30% prevalence depending on ethnic subgroup [10, 11]. The HLA-A serotype group positive (HLA-A*02+) population with HPV+ tumors merits particular focus since the absence of HLA-A*02 correlates with better disease-free survival for patients with HPV+ HNCs [12, 13]. While E6 and E7 have been used in a multitude of programs for therapeutic vaccines to treat HPV+ cancers, there has been no significant progress changing the treatment paradigm for HPV driven cancers [14].

Here we present a Phase 1 study (SQZ-PBMC-HPV-101; NCT04084951) assessing the safety and tolerability of SQZ-PBMC-HPV in patients with advanced-stage, previously treated, HPV16+ solid tumors. Safety results, including dose limiting toxicity (DLT), are included. We report changes in blood cytokines after administration of SQZ-PBMC-HPV, and paired biopsies were characterized for pharmacodynamics effects.

## Methods

### Participants

The study population consists of patients who are HLA-A*02+ with advanced-stage HPV16+ solid tumors. For eligibility HPV16 positivity was determined from circulating free DNA by droplet digital polymerase chain reaction (PCR) analysis or tumor tissue using standard PCR. All eligible patients were retrospectively tested for HPV16 status using PCR. Patients must have progressed after a platinum-based regimen in the adjuvant or recurrent setting. There was no limit to the number of prior lines of treatment a patient could have received. Patients had to have at least one measurable lesion according to Response Evaluation Criteria for Solid Tumors version 1.1 (RECIST 1.1), and a lesion that could be biopsied at screening and on cycle 2, day 8. All subjects had an Eastern Cooperative Oncology Group (ECOG) performance status of 0 to 1. Adequate organ function and bone marrow reserve were assessed by laboratory measurements within 14 days prior to the leukapheresis. No treatment with any anticancer therapy was allowed within 2 weeks of leukapheresis.

Patients who experienced ongoing > Grade 1 adverse events (AEs) according to National Cancer Institute Common Terminology Criteria for Adverse Events (CTCAE V5.0) related to previous treatment with anticancer or investigational therapy that had not resolved (*i*.*e*. ≤ Grade 1) at least 2 weeks prior to leukapheresis were excluded. Subjects who had a history of any Grade 3 immune-related AE (irAE) from prior immunotherapy or any irAE that led to permanent discontinuation of prior immunotherapy were also excluded.

### SQZ-PBMC-HPV investigational product

Participants underwent a single leukapheresis at the study site. The refrigerated leukopak cell concentrate was shipped to the manufacturing site, where the cells were processed using Cell Squeeze^®^ technology, resulting in cytosolic loading of SLPs containing HLA-A*02-restricted E6 and E7 epitopes of HPV16 [8]. The PBMC-HPV cells were then matured with 1 μM CpG 7909, a CpG oligodeoxy-nucleotide [15], for 4 h at 37 °C, and washed prior to formulation. Maturation of the PBMC-HPV with a Toll-Like Receptor 9 Agonist CpG had been previously demonstrated in murine models to optimize in vivo induction of antigen-specific CD8+ T cell responses by squeezed cells. SQZ-PBMC-HPV consists of the drug substance PBMC-HPV, CryoStor^®^ CS10, HypoThermosol^®^ FRS, and human serum albumin. Autologous SQZ-PBMC-HPV was administered via syringe upon thawing, at approximately 10 mL/min.

### Study design and dosing

**Fig. 1:**
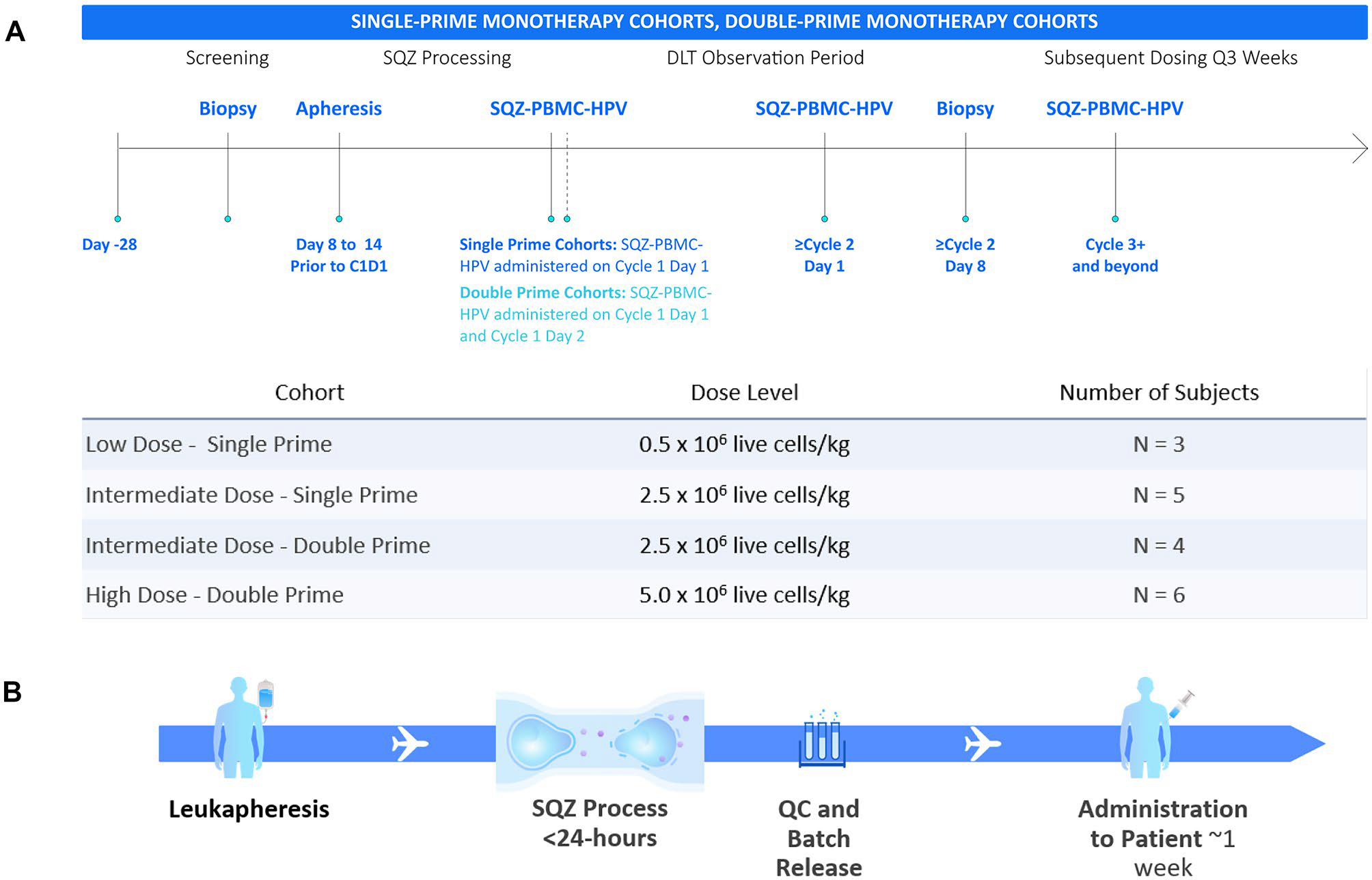
**A** Schema and study cohorts for safety evaluation of SQZ-PBMC-HPV. The observation period for Dose Limiting Toxicity concluded 28 days after the first dose (cycle 2 day 7). For Double Prime cohorts, a second priming dose was administered on cycle 1 day 2; ensuing boosts were single doses. SQZ-PBMC-HPV continued to be administered at 3-week intervals until exhaustion of the subject’s autologous supply of SQZ-PBMC-HPV, treatment discontinuation were met (see Supplemental Material), or for a maximum of 1 year. Disease progression was evaluated per RECIST1.1 criteria, with consideration of the immune confirmed progression according to iRECIST. **B** Process flow for preparation of autologous SQZ-PBMC-HPV, from patient PBMCs collected by leukapheresis

Patients were evaluated in a modified 3+3 design. The Low Dose – and Intermediate Dose – Single Prime cohorts respectively received 0.5 × 10^6^ live cells/kg and 2.5 × 10^6^ live cells/kg of SQZ-PBMC-HPV, as a single priming dose (cycle 1) and received additional administrations on day one of subsequent 21-day cycles (Fig. 1A). The Intermediate Dose – Double Prime cohort received SQZ-PBMC-HPV (2.5 × 10^6^ live cells/kg) on days 1 and 2 of cycle 1 (double priming) and received a single administration on day 1 of ensuing cycles. The High Dose – Double Prime cohort received 5 × 10^6^ live cells/kg on days 1 and 2 of cycle 1 and then a single administration on day 1 of ensuing cycles. Patients were premedicated with 650 mg acetaminophen orally and 25 mg diphenhydramine IV or orally 30 to 60 min before administration of SQZ-PBMC-HPV.

Patients needed to have sufficient autologous drug product to achieve at least 3 full SQZ-PBMC-HPV dose administrations in the assigned cohort, with contingency to be assigned to a lower dose cohort. SQZ-PBMC-HPV was administered in three-week cycles for a maximum of 1 year or until discontinuation criteria was met (see Supplemental Material). Patients were monitored for the occurrence of DLTs for 28 days after the first dose. Per protocol, DLTs were events related to SQZ-PBMC-HPV including any grade 5 toxicity, grade 4 non-hematologic toxicity, grade 3 non-hematologic toxicity that did not improve to grade 1 or baseline within 7 days, grade 4 anemia, grade 4 neutropenia, grade 4 thrombocytopenia, grade 3 thrombocytopenia that did not resolve within 7 days, and grade 3 febrile neutropenia. In all cohorts, patients who experienced disease progression per RECIST 1.1 could continue dosing if considered in their best interest by the treating Investigator to allow for confirmation of disease progression [16].

### Monitoring and assessment

Patient visits included physical exams, recording of vital signs, ECOG evaluation and ECG measurement. Tumor imaging was performed at Screening and every 9 weeks (first year) or every 12 weeks (thereafter), as assessed by the investigator per RECIST 1.1. The first 2 participants in a cohort underwent 23 h of observation after the first administration of SQZ-PBMC-HPV. All patients were observed for at least 4 h after each SQZ-PBMC-HPV administration. A blood sample for cytokine analysis was collected 30 min pre-administration and serially during the observation. Blood draws for cytokine analysis and other pharmacodynamic assessments were also taken at all subsequent visits.

AEs were assessed using CTCAE version 5.0. AE of special interest (AESIs) included events suggestive of hypersensitivity, cytokine release syndrome (CRS), systemic inflammatory response syndrome, influenza-like illness, infusion-related reaction (IRR), and irAEs. In all cohorts, AEs and AESIs that developed after any administered dose had resolved to < Grade 2 at the time of subsequent administration.

A baseline tumor biopsy was obtained within 28 days of the patient’s leukapheresis, and a second biopsy was collected at cycle 2 day 8. Tumor biopsies were analyzed by immunohistochemistry and/or other related techniques in a CAP/CLIA-certified laboratory using both Ventana and Leica autostainers. Brightfield and fluorescent images were digitally captured from microscope slides and analyzed by digital image analysis (HALO or Visiopharm) or Pathologist scoring. The antibodies or probes were used to identify biomarkers and changes in tumor microenvironment. Additional details for the biopsy analyses are provided in the Supplemental Methods. Blood cytokine analysis was also performed in a CLIA-certified laboratory using a Luminexbased multiplexed assay evaluating 11 cytokines (IL-1β, IL-2, IL-4, IL-5, IL-6, IL-8, IL-10, IL-12, IL-13, TNF-a, IFN-γ) and one cytokine receptor (IL-2r).

Further details of the protocol are provided in the Supplementary Methods. The data cut for this report is October 8, 2021. The ClinicalTrials.gov identifier for this multi-phase study is NCT04084951 (https://www.clinicaltrials.gov/ct2/show/NCT04084951).

### Ethics approval and consent to participate

The study was performed in accordance with ethical principles that have their origin in the Declaration of Helsinki and are consistent with the International Conference on Harmonization/Good Clinical Practice and applicable regulatory requirements. The protocol was approved by Institutional Review Boards (IRBs)/Independent Ethics Committees (IECs) at each center. Patients provided written informed consent to participate.

### Data availability

The full data generated in this study are not publicly available due to considerations of patient privacy and consent. Investigators seeking access to specific data may submit a reasonable request to SQZ Biotechnologies (disclosures@sqzbiotech.com).

## Results

### Patient population and disease characteristics

This report covers the enrollment period from December 19, 2019 to October 8, 2021. Table 1 baseline demographics and disease characteristics. Of 84 consented individuals, 18 individuals met eligibility criteria. The majority of consented patients did not meet the HLA*A02 genotyping and/or the HPV16+ criteria. The median age for the participants was 60 (range 50–68). Nine of the patients (50%) had originally presented with a primary HNC; cervical cancer was the site of the primary tumor in only two participants. All subjects had metastatic disease, with 16 (88.9%) presenting metastases in either the lung and/or liver. Patient were heavily pretreated (median of 4 prior regimens); 17 had received immune checkpoint inhibitors (ICIs), with 12 found to be refractory to the ICI treatment.

### Manufacture of SQZ-PBMC-HPV

The median manufacturing time was 17 h, and the vein-to-vein interval ranged 1–2 weeks (Fig. 1B). The median cell viability was 91% across all patient lots. For participants in the highest dose cohort, a median of 4 doses were administered from a single leukapheresis. For all participants, the manufacturing process produced at least three doses.

### Patient exposure

Figure 1A shows the schema for dose escalation cohorts. Three patients were treated in the starting cohort at a dose of SQZ-PBMC-HPV of 0.5 × 10^6^ live cells/kg (Low Dose-Single Prime). The intermediate dose of 2.5 × 10^6^ live cells/kg was explored in the single priming regimen in five patients (Intermediate Dose – Single Prime) and subsequently in the double priming regimen (Intermediate Dose – Double Prime) in four patients. Lastly, six patients were treated in the High Dose – Double Prime cohort, receiving doses of 5 × 10^6^ live cells/kg (Fig. 1A). In the double priming regimens, SQZ-PBMC-HPV was administered on cycle 1 days 1 and 2 separated.

Patients were monitored for the occurrence of DLTs for 28 days after the first dose of SQZ-PBMC-HPV. Administration in subsequent cohorts did not begin until the Study Safety Committee had reviewed the available safety data and determined that dose escalation was warranted. All patients in the safety population were DLT evaluable. The median number of doses administered was 4 (range 2, 10). Five patients completed the treatment when their supply of autologous product was exhausted; nine patients discontinued treatment due to progressive disease; one patient died while on treatment due to an unrelated adverse event and one patient withdrew consent. Supplementary Table S1 and Fig. S1 – provide the full disposition of study subjects as of the cutoff date; two patients were on active treatment at the time of the data cutoff.

### Safety

Of the related treatment emergent adverse events (TEAEs), 93% of the events were Grade 1 or 2. A single occurrence of anemia was the only related Grade 3 TEAE; this occurred in a patient with Grade 2 anemia at baseline. Fatigue was the most common TEAE, reported in 6 participants, with 5 such AEs gauged as treatment related TEAEs (*cf*. Table 2A; see Supplementary Table S2 - for occurrence of all TEAEs). 78% of patients experienced a related TEAE (Table 2B; Supplementary Table S3 provides all related TEAEs apportioned by grade). Treatment related TEAEs occurring in ≥ 15% of the participants were fatigue, flushing, and hypotension.

One participant in the Low Dose – Single Prime cohort experienced a Grade 2 CRS following the second administration of SQZ-PBMC-HPV. This was also the only treatment related serious adverse event (SAE). The CRS was treated with short term oxygen support and hydromorphone, and the individual was hospitalized (< 24 h) for monitoring. The CRS proved self-limiting within hours and the patient became afebrile and hemodynamically stable. No AESIs were reported following the third administration of SQZ-PBMC-HPV.

### Peripheral cellular and cytokine analyses

**Fig. 2:**
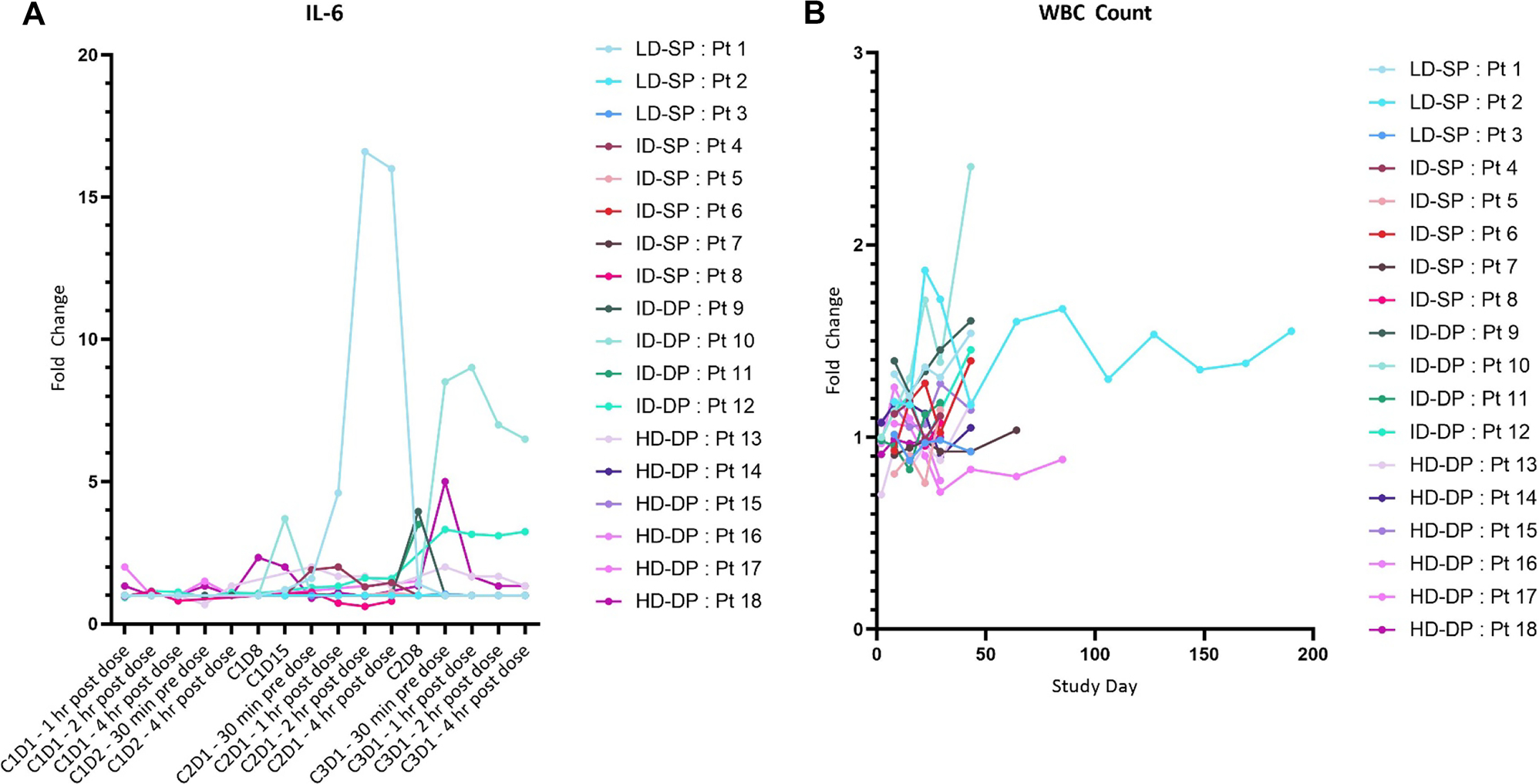
SQZ-PBMC-HPV is not associated with systemic inflammation. **A** Serum IL-6 levels of subjects receiving SQZ-PBMC-HPV. Graph reports relative fold changes of IL-6 compared to the baseline sample drawn 30 min before cycle 1 day 1 administration. Baseline samples were all within accepted range of normal. X-axis is by patient visit and is not linear. **B** White blood cell counts sampled during visits involving physical exams. Note: all patients received scheduled doses of SQZ-PBMC-HPV for cycles 1 and 2, then differed in duration of treatment thereafter. LD-SP: Low Dose – Single Prime; ID-SP: Intermediate Dose – Single Prime; IP-DP: Intermediate Dose – Double Prime; HD-DP: High Dose – Double Prime

Multiplex cytokine analysis was performed on serum samples collected before treatment and serially during observation following infusion of SQZ-PBMC-HPV (Fig. 2A). The Grade 2 CRS was associated with a moderate increase in interleukin 6 (IL-6) from 6 pg/mL at baseline to 83 pg/mL on cycle 2 day 1 (upper limit of normal: 5 pg/mL); thereafter the level returned to the normal range with no recurrence of elevated cytokines. Other than the single described instance of CRS, the monitoring of other serum cytokines as typified by IL-2, gave no indication of elevated systemic inflammation; similarly, there were no significant fluctuations of peripheral white blood cell counts (Fig. 2B; Supplementary Fig. S2).

### Radiographic assessment and intratumoral pharmacodynamics

Of the 18 treated patients, 17 were evaluated for response. Four patients had stable disease as their best response, and one patient (#17) had a best overall response of unconfirmed partial response (Supplementary Fig. S3). Due to the reduction in volume of the target lesion, Patient #17’s dysphagia caused by the target lesion improved from grade 3 to grade 2.

**Fig. 3:**
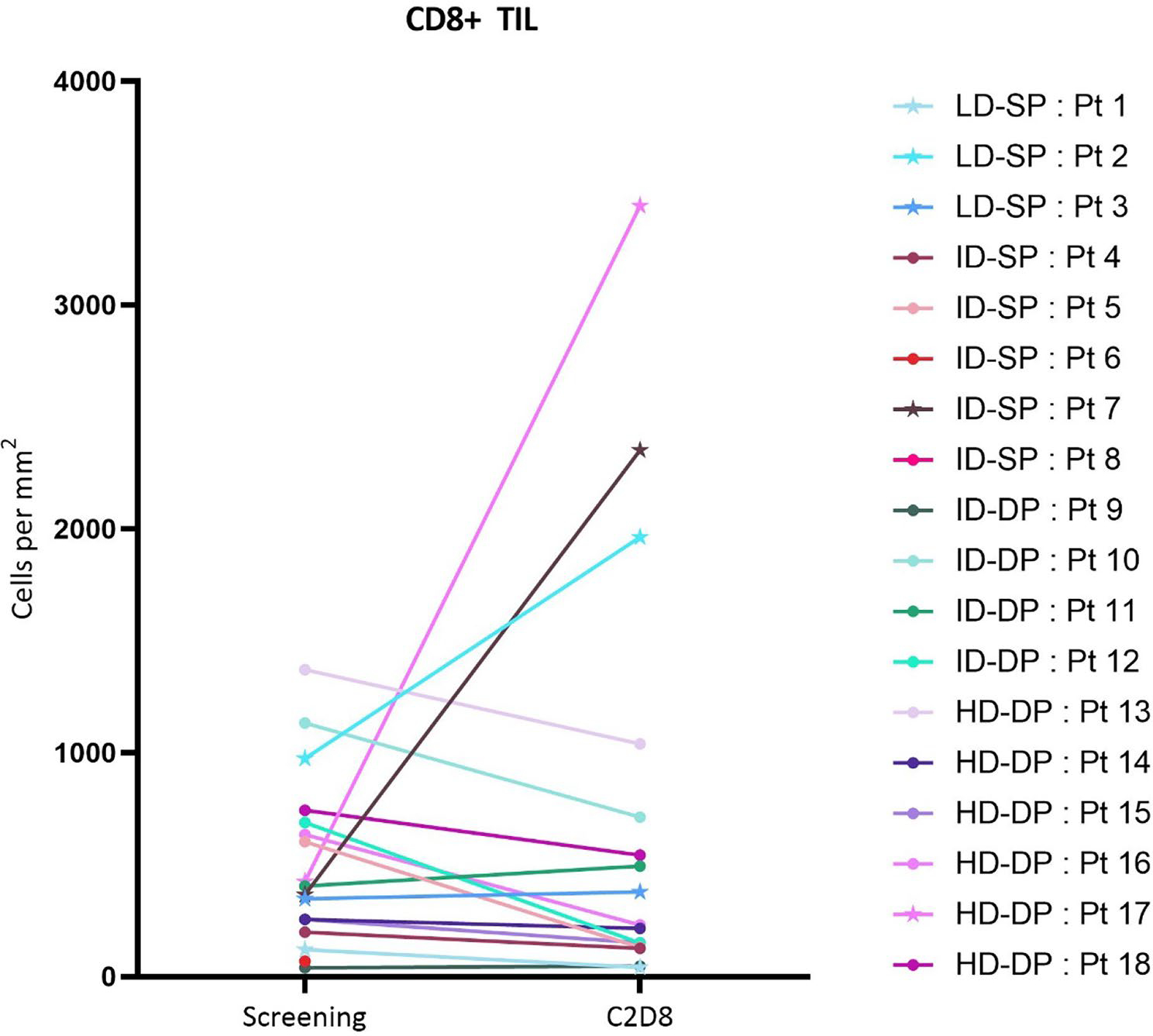
CD8+ tumor infiltrating lymphocytes, as determined by histology analyses using tumor biopsy samples collected during subject screening (within 28 days of first treatment) and at day 28 (cycle 2 day 8) following the first infusion of SQZ-PBMC-HPV. Cohort abbreviations as in Fig. 2. Data represented with a star indicates that the patient had a best overall response of stable disease or better

**Fig. 4:**
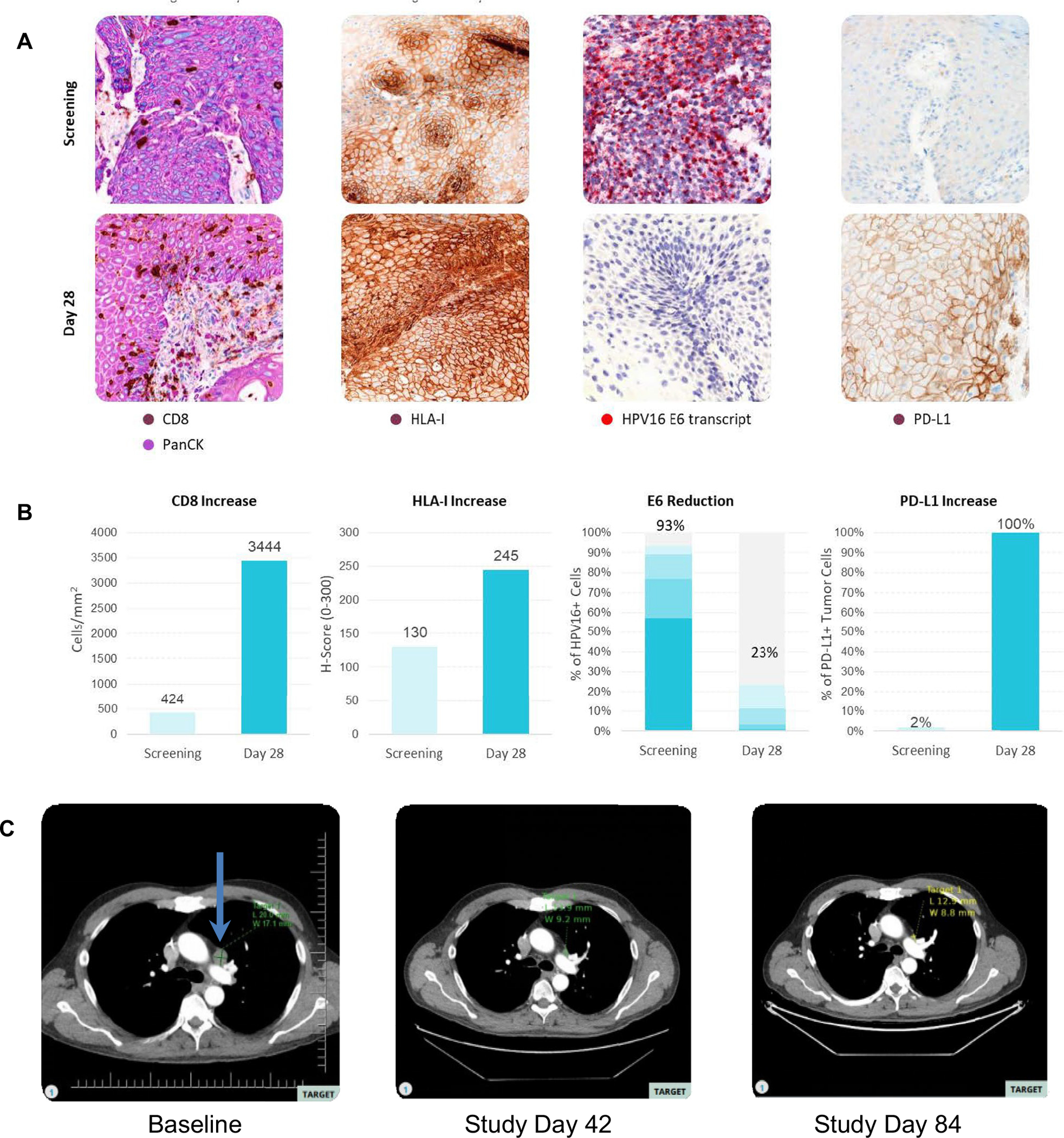
Imaging results and pharmacodynamic parameters for patient 17. **A** Histological evaluation of the indicated analytes. CD8 and PD-L1 by immunostaining; HLA-I and HPV E6 transcript by ISH. The baseline screening biopsy was collected within 28 days prior to the first infusion of SQZ-PBMC-HPV. The second biopsy was collected 28 days after the first treatment. **B** Quantitative analyses of the analytes in corresponding histology images from (**A**). **C** Imaging results for the target lymph node tumor at baseline, and two follow -up images. The tumor that experienced regression is indicated by an arrow in the baseline image. The subject is in the High Dose – Double Prime cohort receiving infusions of 5 × 10^6^ cells/kg

Seventeen paired biopsy samples were examined. The tumor microenvironment (TME) was histologically graded for inflammation phenotype, and changes were assessed in CD8+ cell density, FoxP3+ cell density, CD8+/GZMB+ cell density, HPV16 E6 and E7 transcript levels, PD-L1 and MHC-I presence on tumor cells (Figs. 3 and 4; Supplementary Figs. S4–S9). Overall, there were no significant variations across the entire cohort. However, in three patients, immunohistochemistry documented an increase in the CD8+ TILs in the post-treatment tumor tissue (Fig. 3). These 3 patients also showed an increase in FoxP3+ cell density (Supplementary Fig. S4). The comparison of immunodynamic events and efficacy showed that all three patients with significant increases in CD8+ and FoxP3+ TILs also demonstrated a best overall response of stable disease or better.

For patient 17 in the High Dose - Double Prime cohort, the influx of CD8+ cells observed in the biopsy coincided with a change of the immunophenotype from desert to inflamed [17]. Additionally, there was an increase in density for CD8+ GZMB+ cells and increased levels of MHC-1 expression. There was also a significant reduction in the frequency of cells positive for the transcript of HPV16 E6; E7 expressing cells followed a similar pattern. PD-L1+ tumor cells increased from 2 to 100% (Fig. 4A, B, and Supplementary Figs. S5–S9). The patient also demonstrated a symptomatic improvement in the dysphagia caused by the primary pharyngeal tumor; however, this tumor had an infiltrative nature and was deemed non-measurable for RECIST 1.1 assessment. Evaluation of the target lesion showed a 50% reduction in tumor volume, and the overall response was an unconfirmed partial response (Fig. 4C). Despite continued symptomatic improvement, patient 17 subsequently presented with a new lesion, consistent with progressive disease.

## Discussion

This first-in-human study has documented that SQZ-PBMC-HPV is safe and confirmed the manufacturing feasibility of autologous cell therapies using microfluidic squeezing.

In devising the dosing strategy, we considered information for an approved immunomodulating, autologous cellular therapy in patients with advanced malignancies with a dose of ~0.7 × 10^6^ live monocytes/kg in a 70 kg patient [18]. We selected a slightly lower value of 0.5 × 10^6^ live cells/kg for the Low Dose – Single Prime cohort, with the added proviso that essentially all the cells in SQZ-PBMC-HPV are capable of presenting the loaded antigens, not just the fraction of monocytes. The next higher dose evaluated was predicated on our initial experience conducting the Cell Squeeze^®^ process at a manufacturing scale; based on this prior work there was a good likelihood of obtaining three doses per patient leukapheresis, at 2.5 × 10^6^ live cells/kg [13]. Configuring the double priming regimens was premised on the reported half-lives for MHC-I complexes of immunogenic peptides ranging from 10 – 24 h [19]. These cohorts, with SQZ-PBMC-HPV administrations on cycle 1 days 1 and 2, separated by at least 16 h, should experience a prolonged interval of initial antigen presentation. In practice, manufacturing could accommodate dose escalation to 5 × 10^6^ live cells/kg in the High Dose – Double Prime cohort. All patients had sufficient drug product from a single leukapheresis, and never had to deescalate a participant due to insufficient autologous product. While the current study protocol only allows for a single leukapheresis, additional leukaphereses may be implemented once proof-of-concept has been established.

We observed a favorable safety profile at all dose levels. No DLTs were observed in any cohort. The majority of related TEAEs were Grade 1 or 2. There was one case of a Grade 2 CRS for a subject in the Low Dose – Single Prime cohort. In this patient, medication and supportive therapies led to their quick resolution and the CRS proved self-limiting. Interestingly, the same patient did not report any AEs following the third SQZ-PBMC-HPV administration. While this Grade 2 CRS was associated with an increase in IL-6 on the day of infusion, the overall study exhibited little variation in serum cytokines during the interval on treatment, indicative of no appreciable systemic inflammation [20, 21]. The favorable safety profile applied to both the Single Prime and Double Prime cohorts. Based on these observations, we selected a recommended Phase 2 dose (RP2D) of 5.0 × 10^6^ cells/kg, administered with double priming. The observed unconfirmed partial response in a patient showing dysphagia improvement with shrinkage on a target lesion, and several patients with extended disease control is noteworthy in this heavily pretreated patient population.

The hypothesized mechanism of action (MOA) for SQZ-PBMC-HPV is based upon the previously referenced studies using a murine prototype. Cytosolic loading of patient PBMCs with SLPs containing HLA-A*02-restricted E6 and E7 epitopes of HPV16 results in these cells presenting the aforementioned epitopes in an MHC-I context. Prior studies have shown this cytosolic loading of APCs is 1000 × more potent in eliciting CD8+ T cell responses via MHC-I presentation compared to the process of “cross-presentation” [8, 22]. Following administration, SQZ-PBMC-HPV cells are expected to migrate to lymphoid organs (lymph nodes and spleen), present E6 and E7 to stimulate HPV16-specific CD8+ T cells, which could infiltrate tumors and kill HPV16-expressing tumor cells. Moreover, all cell types within the patient PBMCs undergo cytosolic loading, and are consequently capable of presenting the epitopes. This latter feature led to our hypothesis that even the lowest dose of SQZ-PBMC-HPV could be biologically active, a point demonstrated in the observed increases in tumor infiltrating CD8+ cells for a participant in the Low Dose – Single Prime cohort who experienced clinical benefit. This patient remained on treatment for approximately nine months.

Enhancement of the host cellular immune response against the HPV16+ tumor would result in increased CD8+ T cell density in the TME, as we observed in several patients (Fig. 3). A fuller complement of pharmacodynamic changes were observed in patient 17, who experienced diminished dysphagia from the primary pharyngeal tumor, and where the target lesion underwent an ~50% reduction. Analyses of the day 28 biopsy showed increased CD8+ and FoxP3+ cell densities and an increase in CD8+ GZMB+ T cells in conjunction with increased MHC-I (HLA-A) expression characterizing a shift in the tumor microenvironment to an inflamed phenotype conducive to tumor cell killing rather than immune evasion. Furthermore reduction in the observed frequency of cells positive for E6 and E7 transcripts with the remaining E6- or E7-positive cells showing lower transcript signal intensities suggests an expression profile consistent with an HPV16 E6 and E7 targeted anti-tumor response (Fig. 4).

Observing pharmacodynamic features consistent with the MOA in a subset of individuals leads us to speculate about the cellular and pharmacodynamic outcomes in the other patients. As the comparative biopsies were collected 28 days into treatment, it is possible this did not provide adequate time for similar shifts to manifest, and/or requiring more treatment cycles in some participants. This may reflect the state of CD8+ T cell exhaustion and/or dysfunction that attends persistent exposure to tumor and viral antigens [23, 24]. The knowledge accrued from continued enrollment in the High Dose – Double Prime cohort may shed light on the characteristics of patients who respond well to administration of SQZ-PBMC-HPV monotherapy, possibly indicative of this state of immune exhaustion. The mechanisms implicated in the resistance to anti-cancer vaccines can be divided into intrinsic and extrinsic mechanisms [25]. The intrinsic mechanisms can be classified into the mutations in signaling pathways supporting tumor-immune control, the loss of tumor antigen expression, the changes in antigen processing pathways, and the loss of HLA expression, epigenetic changes, and increased expression of immunosuppressive ligands [26, 27]. The extrinsic mechanisms of resistance are those associated with a cold tumor microenvironment characterized by the presence of immunosuppressive cells (such as CAFs, MDSCs, Tregs, and M2 macrophages) and immunosuppressive cytokines that can inhibit the activation of CD8+ T cells directly or indirectly. The combination of vaccine approaches with checkpoint inhibitors may offer synergies to overcome the extrinsic mechanisms of resistance [28].

A critical observation for the response experienced by patient 17 was the change in PD-L1 expression, from 2 to 100% of cells. Certainly, engagement of the PD-1 pathway would dampen the activity of tumor-infiltrating lymphocytes, the PD-1 pathway being one avenue exploited by tumors to evade the immune system [17]. This leads to the proposal of combining SQZ-PBMC-HPV with ICIs, a manner of synergy already demonstrated in murine models [29], and such cohorts are currently enrolling.

The short vein-to-vein interval (1 – 2 weeks) derives in part from the simplicity of the microfluidic squeezing process whereby product manufacturing is accomplished in < 24 h; the rest of the interval is comprised of materials transit, release testing and clinic scheduling. This is in contrast to the complexity and duration in the manufacture of other autologous adoptive cellular therapies that require genetic manipulations (*e*.*g*. engineered TCR T cells, CAR T cells) and even those that don’t involve genetic engineering such as antigen-loaded APCs [30] and TILs [31–36]. Additionally, patients are not subjected to lymphodepletion or preconditioning, further simplifying treatment. As a corollary to the simplicity of manufacturing, we are presently researching techniques to implement Cell Squeeze^®^ systems at point-of-care that would streamline logistics and further accelerate the time to delivery of autologous cell therapy [37].

In conclusion, we report safety and encouraging indications of activity for SQZ-PBMC-HPV in patients with advanced metastatic, HPV16+ solid tumors, observing increases in CD8+ TILs for several patients; the latter are strongly prognostic of improved outcomes [38]. The simplicity of the method allows for extension to other cancer types, targeting antigens specific to other transforming viruses or to tumor neoantigens. From ongoing monotherapy studies, we will continue to learn how to stratify prospective patients as to the likelihood of responding to SQZ-PBMC-HPV, including discerning attributes of immune exhaustion or senescence that could blunt potential vaccine responses. Likewise we are testing SQZ-PBMC-HPV with ICIs, seeking superior response rates and improved disease outcomes.

## Supplementary Material

1

2

3

## Figures and Tables

**Table 1:** Patient demographics and disease characterization for patients enrolled in SQZ-PBMC-HPV-101 (NCT04084951)

**Patient Demographics** ^a^	
Total Consented (n)	84
Total Enrolled (n)	18
Median Age (Min, Max)	60 (47, 78)
Sex	50%/50% M/F
**Ethnicity**	
Hispanic or Latino	2 (11.1%)
Caucasian or Not Reported	16 (88.9%)
**Disease Characteristics**	
**Primary Tumor Site (all classed squamous cell)**	
Anus	7 (38.9%)
Cervix	2 (11.1%)
Head and neck	9 (50.0%)
Oropharynx	5 (55.6%)
Tongue	3 (33.3%)
Neck	1 (11.1%)
**Time Since Diagnosis (months)**	
Median	44
Min, Max	12, 80
**Liver Metastasis at Baseline**	
Yes	9 (50.0%)
No	8 (44.4%)
Unknown	1 (5.6%)
**Lung Metastasis at Baseline**	
Yes	10 (55.6%)
No	7 (38.9%)
Unknown	1 (5.6%)
**Prior Regimens**	
Median (Min, Max)	4 (1, 7)
**Prior Treatments**	
Prior platinum-based chemotherapy	17 (94.4%)
Prior checkpoint immunotherapy	17 (94.4%)
Prior radiation	17 (94.4%)
Prior surgery	12 (66.7%)

a Detailed tables with breakdown by treatment cohort appear in the supplemental materials

**Table 2:** Treatment Related TEAEs.

**Section A.**					

**MedDRA Preferred Term**	**Low Dose – Single Prime** 0.5 × 10^6^ live cells/kg (n = 3)	**Intermediate Dose – Single Prime** 2.5 × 10^6^ live cells/kg (n = 5)	**Intermediate Dose – Double Prime** 2.5 × 10^6^ live cells/kg (n = 4)	**High Dose – Double Prime** 5.0 × 10^6^ live cells/kg (n = 6)	**Total** (N = 18)

Any Treatment Related TEAE	3 (100.0)	4 (80.0)	2 (50.0)	5 (83.3)	14 (77.8)
Fatigue	0	1 (20.0)	0	4 (66.7)	5 (27.8)
Flushing	1 (33.3)	0	0	2 (33.3)	3 (16.7)
Hypotension	2 (66.7)	1 (20.0)	0	0	3 (16.7)
Infusion related reaction	1 (33.3)	0	1 (25.0)	0	2 (11.1)
Nausea	0	0	0	2 (33.3)	2 (11.1)
Pruritus	0	1 (20.0)	0	1 (16.7)	2 (11.1)
Anaemia	0	1 (20.0)	0	0	1 (5.6)
Blood creatinine increased	0	0	0	1 (16.7)	1 (5.6)
Chills	1 (33.3)	0	0	0	1 (5.6)
Cough	1 (33.3)	0	0	0	1 (5.6)
Cytokine release syndrome	1 (33.3)	0	0	0	1 (5.6)
Dyspnoea	0	1 (20.0)	0	0	1 (5.6)
Hyponatremia	0	0	1 (25.0)	0	1 (5.6)
Immunisation anxiety related reaction	0	0	0	1 (16.7)	1 (5.6)
Lymph node pain	1 (33.3)	0	0	0	1 (5.6)
Malaise	1 (33.3)	0	0	0	1 (5.6)
Myalgia	0	0	0	1 (16.7)	1 (5.6)
Nasal congestion	1 (33.3)	0	0	0	1 (5.6)
Pancytopenia	0	0	0	1 (16.7)	1 (5.6)
Rash pruritic	0	0	0	1 (16.7)	1 (5.6)
Weight decreased	0	0	0	1 (16.7)	1 (5.6)
Wheezing	1 (33.3)	0	0	0	1 (5.6)

**Section B.**					
**Number of Patients with: [n (%)]**	**Low Dose – Single Prime** 0.5 × 10^6^ live cells/kg (n = 3)	**Intermediate Dose – Single Prime** 2.5 × 10^6^ live cells/kg (n = 5)	**Intermediate Dose – Double Prime** 2.5 × 10^6^ live cells/kg (n = 4)	**High Dose – Double Prime** 5.0 × 10^6^ live cells/kg (n = 6)	**Total (N = 18)**

Related TEAE	3 (100.0)	4 (80.0)	2 (50.0)	5 (83.3)	14 (77.8)
Grade 1	1 (33.3)	2 (40.0)	1 (25.0)	4 (66.7)	8 (44.4)
Grade 2	2 (66.7)	1 (20.0)	1 (25.0)	1 (16.7)	5 (27.8)
Grade 3	0	1 (20.0)	0	0	1 (5.6)
Grade 4	0	0	0	0	0
Grade 5	0	0	0	0	0

A. The number of instances of related TEAEs by the MedDRA Preferred Term.

B. Summary of the related TEAEs by Grade

## Data Availability

The full data generated in this study are not publicly available due to considerations of patient privacy and consent. Investigators seeking access to specific data may submit a reasonable request to SQZ Biotechnologies (disclosures@sqzbiotech.com).

## Statement of translational relevance

Human papilloma virus (HPV) is the most common sexually transmitted infection in the United States, causing ~44,000 cases of anal, cervical, oropharyngeal, penile, vaginal, and vulvar cancers yearly in the United States. Autologous PBMCs were loaded with antigen precursors by microfluidic squeezing, resulting in HPV16 E6 and E7 antigen presentation via MHC-I; the cell mixture consists primarily of T cells, B cells, natural killer cells, and monocytes (SQZ-PBMC-HPV). A dose escalation Phase 1 study in patients with advanced/metastatic HPV16+ cancers documented good tolerability and defined the dose for future studies, as well as the overall feasibility of the manufacturing process. In addition, there was one unconfirmed response, and the tumor immune contextual data supported the hypothesized mechanism of action. Additional research in combination with other immune therapies is ongoing to assess the potential impact of this therapy in patients with HPV16+ cancers.
