# Supplementary material for: Phase 1 study to determine the safety and dosing of autologous PBMCs modified to present HPV16 antigens (SQZ-PBMC-HPV) in HLA-A*02+ patients with HPV16+ solid tumors
Source: Invest New Drugs. Author manuscript; Available in PMC 2024 Apr 1. (PMC10140074; doi:10.1007/s10637-023-01342-x)
Supplement: 1 [file NIHMS1895206-supplement-1.docx]

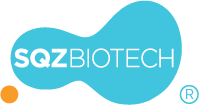


Supplemental methods accompanying:

**Phase 1 study to determine the safety, tolerability, and recommended dose of**

**autologous PBMCs modified for antigen presentation targeting HPV16**

**(SQZ-PBMC-HPV) in HLA-A*02+ patients with HPV16+ Solid Tumors**

**Sections**

Section 1 Protocol Synopsis

Section 2 Other Protocol Particulars

Section 3 Product Testing and Histochemistry Methods

Section 4 References

**Tables**

Table 1 Schedule of Assessments: SQZ-PBMC-HPV Monotherapy, Dose Escalation.

Table 2 Responsibilities of the SSC and DSMB

Table 3 Management of Infusion-Related Reactions

Table 4 Management of Cytokine Release Syndrome

1. PROTOCOL SYNOPSIS

| Protocol Title | A Phase 1/2, Multicenter, Open-Label, Dose-Escalation and Dose-Expansion Study of SQZ‑PBMC‑HPV as Monotherapy and in Combination with Atezolizumab or Other Immune Checkpoint Inhibitors in HLA‑A*02+ Patients with HPV16+ Recurrent, Locally Advanced or Metastatic Solid Tumors |
| --- | --- |
| Study Number | SQZ‑PBMC‑HPV‑101 |
| ClinTrials.gov Registration Number | NCT04084951 |
| Development Phase | Phase 1/2 |
| Sponsor | SQZ Biotechnologies |
| Study Centers | Multicenter, up to 20 sites |
| Number of Patients | 18 patients were enrolled in the Phase 1 evaluation, as of the data closure date of 10/08/2021 for this publication. |
| Study Objectives | **Primary Objectives**  **Part 1 (Monotherapy Dose Escalation Phase):**  To characterize the safety and tolerability of SQZ‑PBMC‑HPV administered as monotherapy to human leukocyte antigen serotype within the HLA-A serotype group positive (HLA-A*02+) patients with human papillomavirus (HPV) type 16 (HPV16) positive (HPV16+) recurrent, locally advanced, or metastatic solid tumors  To determine the recommended Phase 2 dose (RP2D) of SQZ‑PBMC‑HPV monotherapy  **Secondary Objectives**  To assess the antitumor activity of SQZ‑PBMC‑HPV in patients with recurrent, locally advanced, or metastatic solid tumors  To assess the manufacturing feasibility of SQZ-PBMC-HPV  **Exploratory Objectives**  To explore changes in blood cytokines after treatment with SQZ‑PBMC‑HPV  To characterize the immunogenic and pharmacodynamic effects (on selected pharmacodynamic parameters) and duration of pharmacodynamic response following SQZ‑PBMC-HPV administration  To explore blood and/or tumor genomic markers in consenting patients receiving SQZ‑PBMC‑HPV |
| Study Design | This is a Phase 1/2, open-label, multicenter study of the safety and tolerability, antitumor activity, and immunogenic and pharmacodynamic effects of SQZ‑PBMC‑HPV as monotherapy in HLA‑A*02+ patients with recurrent, locally advanced, or metastatic HPV16+ solid tumors.  The study population consists of patients who are HLA-A*02+ with advanced-stage HPV16+ solid tumors (head and neck, cervical cancer, and other tumor types). HLA‑A*02+ status and HPV16+ tumor status must be confirmed via laboratory reports, and all eligibility criteria must be met prior to the patient’s leukapheresis. Patients with locally confirmed HPV16+ status may have central confirmation done from the fresh tumor biopsy collected at Screening if documentation of laboratory accreditation is deemed by the Sponsor to be insufficient.  Eligible patients will undergo a single leukapheresis at the study sites. The leukapheresis product will be sent to the contract manufacturer for manufacture of each patient’s personalized autologous cellular therapy. Frozen vials of SQZ‑PBMC‑HPV will then be sent to the study sites for administration.  Part 1 consists of dose escalation to determine the RP2D of SQZ-PBMC-HPV monotherapy.  In all cohorts, SQZ‑PBMC-HPV will be administered at 3‑week intervals until the SQZ‑PBMC‑HPV supply is exhausted, treatment discontinuation criteria are met, or for a maximum of 1 year, whichever comes first. Patients who experience disease progression per Response Evaluation Criteria for Solid Tumors version 1.1 (RECIST 1.1) may continue dosing if considered in their best interest by the treating Investigator to allow for confirmation of disease progression, *i*.*e*. immune confirmed progression (iCPD) according to modified RECIST criteria for incorporation into solid tumor studies of immunotherapeutics (iRECIST) (1).  **Part 1: Escalation Phase (SQZ-PBMC-HPV Monotherapy)**  Patients will be evaluated in a modified 3+3 dose-escalation design. At least 2 dose levels (0.5 × 10P^6^P live cells/kg and 2.5 × 10^6^ live cells/kg) of SQZ‑PBMC‑HPV will be evaluated as monotherapy (Cohorts 1, 2, and 3). Patients in Cohorts 1 and 2 will receive SQZ-PBMC-HPV on Day 1 of each 21‑day cycle (single priming); patients in Cohort 3 will receive SQZ‑PBMC‑HPV on Days 1 and 2 of Cycle 1 (double priming) and Day 1 of each subsequent cycle. In each cohort, the first 2 patients must complete Cycle 1 Day 8 (C1D8) before additional patients in the cohort can be treated in that cohort.  Patients must have sufficient autologous drug product to achieve at least 3 full SQZ‑PBMC‑HPV dose administrations to be dosed in the assigned cohort or will get assigned to a lower dose cohort.  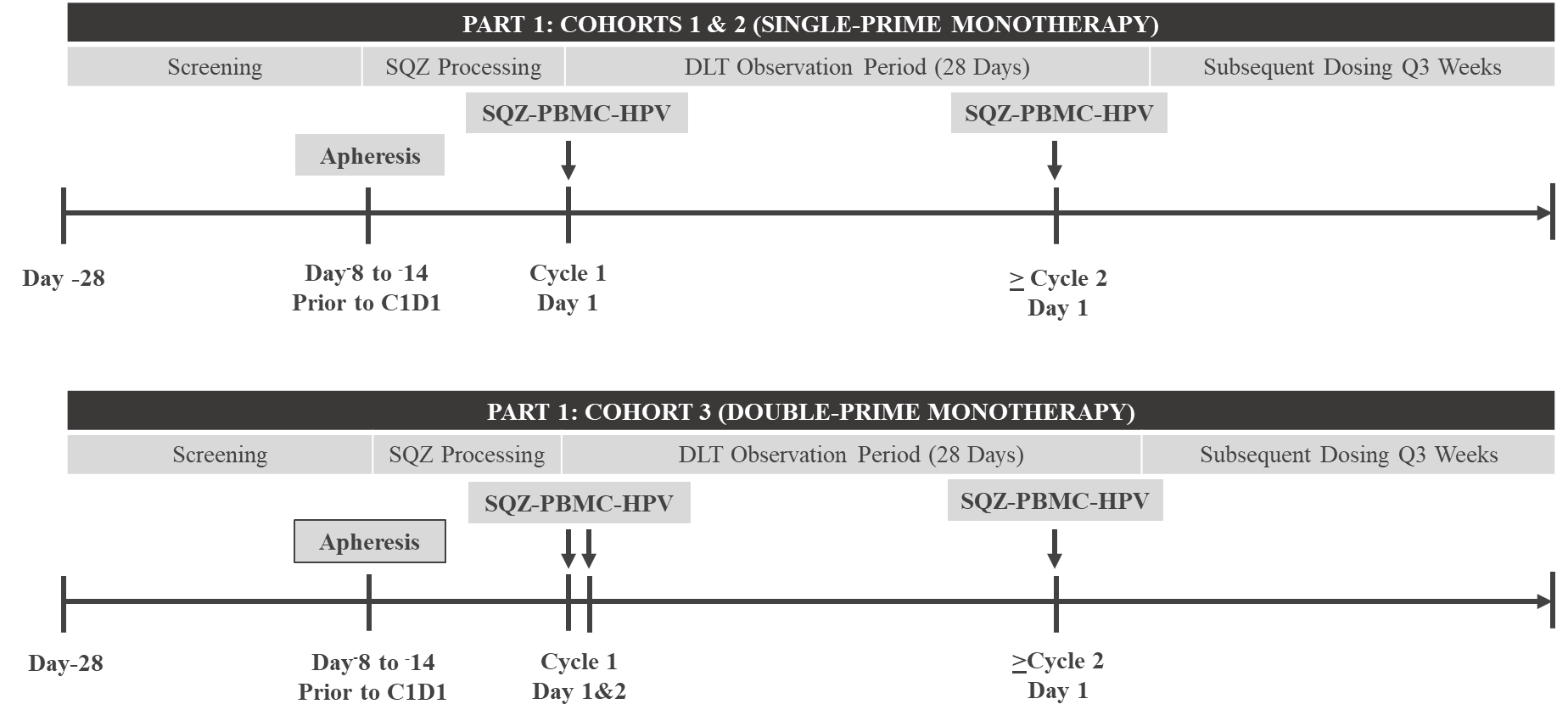**Schema:**  Abbreviations: C1D1=Cycle 1, Day 1; DLT=Dose-Limiting Toxicity; HPV=Human Papilloma Virus; PBMC=Peripheral Blood Mononuclear Cells; Q3W=Every 3 Weeks; SQZ=SQZ-PBMC-HPV.  Patients will be enrolled in a staggered manner across all investigative sites, meaning no more than 1 patient in a cohort will receive the first administration of SQZ‑PBMC-HPV within 1 week. Administration of SQZ‑PBMC-HPV in subsequent cohorts will not begin until the Study Safety Committee (SSC) has reviewed available safety data and determined that dose escalation is warranted. Patients will be monitored for the occurrence of dose‑limiting toxicities (DLTs) for 28 days after the first dose of SQZ‑PBMC-HPV in monotherapy cohorts. Additional patients may be treated in a cohort to further investigate safety and tolerability, immunogenic effects, and antitumor activity. There will be a maximum of 12 patients per cohort.  Following review of the available safety, efficacy, and pharmacodynamic data from patients in Cohort 3, the SSC may determine that exploration of additional higher or lower single- or double‑priming dose levels is warranted. In this case, the magnitude of the dose escalation or de‑escalation will be determined by the SSC based on the type and severity of treatment‑emergent adverse events (TEAEs) observed. Subsequent higher dose cohorts will be denoted 3a or 3b; lower dose cohorts will be denoted 3a-1 or 3b-1. The planned Cohort 3 higher dose (*e*.*g*. 3a) regimens are listed below, but intermediate dose levels may be selected if the SSC deems it necessary based on review of available safety data:  5 × 10^6^ live cells/kg (single prime)  5 × 10^6^ live cells/kg (double prime)  A lower dose level (single or double prime), as determined by the SSC.  Mapping of these Cohort names to those employed in the manuscript:   \| Cohort 1 \| Low Dose – Single Prime \| \| --- \| --- \| \| Cohort 2 \| Intermediate Dose – Single Prime \| \| Cohort 3 \| Intermediate Dose – Double Prime \| \| Cohort 3a \| High Dose – Double Prime \|   For each patient, in all cohorts, TEAEs that develop after any administered dose should have resolved to ≤Grade 2 at time of subsequent administrations. Adverse events of special interest (AESIs) that develop after any administered dose should have resolved to <Grade 2 at time of subsequent administration (whether on Cycle 1 Day 2, Cycle 2 Day 1, or Day 1 of any subsequent cycle). In Cohort 3, if these retreatment criteria are met, the second SQZ‑PBMC‑HPV administration should be given during the ≥23-hour observation period (*i*.*e.* between 16 and 24 hours post first dose). Patients will be observed for a minimum of 4 hours after the second priming administration. The minimum interval between the 2 administrations should be 16 hours.  The RP2D for monotherapy will be selected based on review of all available safety, tolerability, immunogenic, and other pharmacodynamic and antitumor data. For selection of the RP2D for monotherapy, the DLT assessment in all cohorts must be complete. It is possible that the maximum tolerated dose (MTD) cannot be determined; in this case, the maximum administered dose (MAD) will be the RP2D.  **All Patients**  Patients who experience disease progression per RECIST 1.1 may continue dosing if considered in their best interest by the treating Investigator to allow for confirmation of disease progression, *i*.*e.* iCPD according to iRECIST (1, 2).  After the last dose of investigational product, follow-up visits will occur to monitor safety and tolerability and evaluate overall survival (OS). |
| Investigational Product | The investigational product, SQZ‑PBMC‑HPV, consists of autologous peripheral blood mononuclear cells (PBMCs) presenting immunogenic epitopes of the E6 and E7 proteins of HPV16. |
| Treatment Cohorts | All patients should be premedicated with 650 mg acetaminophen and 25 mg diphenhydramine IV or orally (or another H1 antagonist at an equipotent dose) approximately 30 to 60 minutes prior to administration of SQZ‑PBMC‑HPV. A cycle is defined as a treatment period of 21 days.  **Part 1 (Escalation Phase):**  SQZ-PBMC-HPV is administered Q3W. In Cycle 1, patients in the single-prime regimen (Cohorts 1 and 2) will receive SQZ‑PBMC‑HPV on Day 1 and patients in the double‑prime regimen (Cohort 3) will receive SQZ‑PBMC‑HPV on Days 1 and 2 of Cycle 1 and a single dose on Day 1 in subsequent cycles.  Cohort 1: low cell dose of 0.5 × 10^6^ live cells/kg on Day 1 of each 3-week cycle (single prime)  Cohort 2: high cell dose of 2.5 × 10^6^ live cells/kg on Day 1 of each 3-week cycle (single prime)  Cohort 3: high cell dose of 2.5 × 10^6^ live cells/kg on Days 1 and 2 of Cycle 1 (double prime) and on Day 1 in subsequent cycles  Dependent on available safety, efficacy, and pharmacodynamic data, additional cohorts, at a higher or lower dose, may be added. |
| Duration of Treatment | All patients must undergo a single leukapheresis at the study sites; this should typically occur 8 to 14 days prior to the initial administration of SQZ-PBMC-HPV.  SQZ‑PBMC-HPV will be administered at 3‑week intervals until treatment discontinuation criteria are met, until investigational product is exhausted, or for a maximum of 1 year, whichever comes first. |
| Study Population | The study population consists of patients who are HLA-A*02+ with advanced-stage HPV16+ solid tumors (head and neck, cervical cancer, and other tumor types). In Parts 1 and 2, enrollment of HIV positive (HIV+) patients must be discussed with the Sponsor.  Patients may have received prior therapy with a programmed death-1 (PD-1), programmed death‑ligand 1 (PD-L1), or cytotoxic T‑lymphocyte–associated antigen 4 inhibitor (including ipilimumab or any other antibody or drug specifically targeting T‑cell co‑stimulation or checkpoint pathways). |
| Inclusion Criteria | 1. Male or female patients ≥18 years of age who are HLA-A*02+, as confirmed by genotyping assay from blood. 2. Histologically confirmed incurable or metastatic solid tumors (including but not limited to cervical and head and neck tumors) that are HPV16+ as determined by polymerase chain reaction, histological *in situ* hybridization or circulating cell-free deoxyribonucleic acid (DNA) analysis. 3. For cervical cancer, which is not amenable to curative treatment with surgery, radiation, and/or chemoradiation therapy, the cancer must have progressed after prior systemic chemotherapeutic treatment with a platinum-based regimen in the adjuvant or recurrent setting. Patients must have progressive disease while receiving or after the completion of the most recent prior treatment.   For patients who are intolerant to or refuse a platinum-based systemic chemotherapeutic treatment for recurrent disease, reasons must be documented.   1. For recurrent and metastatic head and neck cancer, which is not amenable to curative treatment with surgery, radiation, and/or chemoradiation therapy, the cancer must have progressed following at least 1 prior platinum-based chemotherapy in the primary, adjuvant, or recurrent setting and have been offered checkpoint immunotherapy. Patients who relapsed after platinum-containing definitive chemoradiation or after adjuvant chemoradiation are eligible if a platinum re-challenge at time of relapse is not seen as beneficial.   For patients who are intolerant to or refuse platinum-based chemotherapy for recurrent disease, reasons must be documented.   1. Patients with incurable or metastatic HPV16+ cancers other than cervical or head and neck cancer must have progressed after at least 1 available standard therapy for incurable disease, or the patient is intolerant to or refuses standard therapy(ies) or has a tumor for which no standard therapy(ies) exist.   **Escalation Phase (Part 1)**   - 1. Enrollment of HIV+ patients should be discussed with the Sponsor.  1. Eastern Cooperative Oncology Group (ECOG) performance status (PS) of 0 to 1. 2. Patients must agree to venous access for the leukapheresis and be willing to have a central line inserted if venous access is an issue. 3. Patients with unresectable or metastatic solid tumors must have a lesion that can be biopsied with acceptable clinical risk and agree to have a fresh biopsy at Screening and on Cycle 2 Day 8 (±2 days). 4. A lesion in a previously irradiated area could be biopsied as long as there is objective evidence of progression of the lesion before study enrollment. 5. At least 1 measurable lesion according to RECIST 1.1. 6. A lesion in a previously irradiated area is eligible to be considered as measurable disease if there is objective evidence of progression of the lesion before study enrollment. 7. Adequate organ function and bone marrow reserve as indicated by the following laboratory assessments performed within 14 days prior to the leukapheresis: 8. Bone marrow function: absolute neutrophil count ≥1000/µL; hemoglobin ≥9 g/dL; platelet count ≥75,000/µL. **NOTE:** In stabilized patients with hemoglobin values <9 g/dL, a blood transfusion may be utilized to meet inclusion criterion. 9. Hepatic function: total serum bilirubin ≤1.5 × upper limit of normal (ULN); serum aspartate aminotransferase (AST)/alanine aminotransferase (ALT) ≤2.5 × ULN (≤5 × ULN in the presence of hepatic metastases); and alkaline phosphatase <2.5 × ULN with the following exception: patients with liver and bone involvement: alkaline phosphatase ≤5 × ULN.  - Patients with inherited disorders of bilirubin metabolism should be discussed with the Sponsor.  1. Renal function: serum creatinine ≤2.5 × ULN or creatinine clearance ≥30 mL/min based either on urine collection or Cockcroft-Gault estimation. 2. Coagulation profile: prothrombin time (PT), international normalized ratio (INR)/partial thromboplastin time ≤1.5 × ULN. Patients on a stable, maintenance regimen of anticoagulant therapy for at least 30 days prior to leukapheresis may have PT/INR measurements >1.5 × ULN if, in the opinion of the Investigator, the patient is suitable for the study. An adequate rationale must be provided to the Sponsor prior to enrollment. 3. Patients with immune-mediated endocrinopathies following treatment with immune checkpoint inhibitors requiring hormone replacement therapy are eligible. 4. Patients requiring prednisone as part of hormone replacement therapy are eligible if the daily doses do not exceed 10 mg. 5. Female patients of childbearing potential must: 6. Have a negative serum beta human chorionic gonadotropin pregnancy test at Screening, and 7. Agree to use double contraception from the time of informed consent until at least 5 months after the last dose of immune checkpoint inhibitor or SQZ‑PBMC‑HPV. 8. Male patients who are not vasectomized must be willing to use condoms from the time of informed consent until at least 5 months after the last dose of immune checkpoint inhibitor or SQZ‑PBMC‑HPV. 9. The patient is capable of understanding and complying with the protocol and has signed the required informed consent form (ICF). The appropriate ICF must be signed before relevant study procedures are performed. If applicable, the female partner of a male patient understands and signs the pregnant partner ICF. |
| Exclusion Criteria | 1. Treatment with anticancer therapy, including investigational therapy, within 2 weeks prior to leukapheresis. For prior therapies with a half-life longer than 3 days, timing of discontinuation of the therapy should be discussed with the Sponsor. 2. Patients with >Grade 1 AEs (except Grade 2 neuropathy, ototoxicity, mucositis, fatigue, alopecia, and endocrine disorders managed with hormone replacement) according to National Cancer Institute (NCI) Common Terminology Criteria for Adverse Events (CTCAE) version 5.0 related to previous treatment with anticancer or investigational therapy that do not resolve (*i*.*e.* ≤Grade 1 or better) at least 2 weeks prior to leukapheresis. 3. History of any Grade 3 immune-related AE (irAE) from prior immunotherapy (patients with endocrinopathy managed with replacement therapy or asymptomatic elevation of serum amylase or lipase are eligible), or any irAE that led to permanent discontinuation of prior immunotherapy. 4. Patients treated with noncorticosteroid-based immunosuppressive agents within the last 6 months may not be eligible and should be discussed with the Sponsor. 5. Patients with active, known, or suspected autoimmune disease may not be eligible and should be discussed with the Sponsor. 6. Patients with prior allogeneic bone marrow or solid organ transplantation may not be eligible and should be discussed with the Sponsor. 7. Live virus vaccination within 4 weeks prior to leukapheresis. 8. Systemic treatment with either corticosteroids (>10 mg of prednisone or the equivalent per day) or other immunosuppressive medications within 14 days prior to leukapheresis. Inhaled, intranasal, intra-articular, and topical (including ocular) steroids are allowed. The use of fludrocortisone for mineralocorticoid replacement in patients with adrenal insufficiency is allowed. 9. Has known active central nervous system metastases and/or carcinomatous meningitis. Patients with previously treated brain metastases may participate provided they are stable (without evidence of progression by imaging for at least 4 weeks prior to the first dose of investigational product, and any neurologic symptoms have returned to Baseline), have no evidence of new or enlarging brain metastases, and are not using steroids for at least 7 days prior to leukapheresis. This exception does not include carcinomatous meningitis, which is excluded regardless of clinical status. 10. History of interstitial lung disease requiring steroids, idiopathic pulmonary fibrosis, pneumonitis (including drug induced), or organizing pneumonia (*e*.*g*. bronchiolitis obliterans, cryptogenic organizing pneumonia). 11. Patients with asymptomatic pneumonitis who have not required steroid therapy for pneumonitis are eligible. 12. Clinically significant cardiac disease, including unstable angina, acute myocardial infarction within 6 months prior to leukapheresis, New York Heart Association class III or IV congestive heart failure, and arrhythmia requiring therapy. 13. Systemic arterial thrombotic or embolic events, such as cerebrovascular accident (including ischemic attacks), within 1 month prior to leukapheresis. 14. Systemic venous thrombotic events (*e*.*g*. deep vein thrombosis) or pulmonary arterial events (*e*.*g*. pulmonary embolism) within 1 month prior to leukapheresis. 15. Patients with venous thrombotic events before leukapheresis on stable anticoagulation therapy are eligible. 16. History or presence of an abnormal electrocardiogram (ECG) that, in the Investigator’s opinion, is clinically meaningful. 17. Left ventricular ejection fraction (LVEF) <50%. 18. Major surgery within 2 weeks of leukapheresis; following major surgeries >2 weeks prior to leukapheresis, all surgical wounds must be healed and free of infection or dehiscence. 19. Any other clinically significant comorbidities, such as active infection, known psychiatric or neurological disorder, or any other condition, which in the judgment of the Investigator, could compromise compliance with the protocol, interfere with the interpretation of study results, or predispose the patient to safety risks. 20. Known active hepatitis B virus or hepatitis C virus, or active mycobacterium tuberculosis infection. 21. Patient has history of alcohol and/or illicit drug abuse within 12 months of entry. 22. Female patients who are breastfeeding or have a positive serum pregnancy test at the Screening visit. 23. Patient has a history of allergy or hypersensitivity to any component of SQZ‑PBMC‑HPV. 24. History of severe allergic anaphylactic reactions to chimeric, human, or humanized antibodies or infusion proteins (combination cohorts only). 25. Known hypersensitivity to atezolizumab, ipilimumab, nivolumab, Chinese hamster ovary cell products or any component of the atezolizumab, ipilimumab, or nivolumab formulation (combination cohorts only). |
| Criteria for Evaluation | **Dose-Limiting Toxicities:** A DLT is defined as an AE or abnormal laboratory value assessed by the Principal Investigator and confirmed by the SSC as unrelated to disease, disease progression, intercurrent illness, concomitant medications/procedures, or environmental factors but related to SQZ‑PBMC-HPV (either alone or in combination), occurring within either the first 28 days of treatment with monotherapy or the first 42 days of treatment with combination therapy, and which meets any of the predefined criteria as listed below using CTCAE version 5.0. Grading of cytokine release syndrome (CRS) and neurotoxicity will use the American Society for Transplantation and Cellular Therapy Consensus Grading.  Non-hematologic toxicity   - - Grade 4 or Grade 5   - Grade 3 toxicity that does not resolve to ≤Grade 1 or Baseline within 7 days despite optimal supportive care, except for Grade 3 CRS or neurotoxicity that does not resolve to ≤Grade 2 within 24 hours   - Grade 3 laboratory value that persists >7 days and requires medical intervention   - >Grade 3 hepatic toxicity lasting >48 hours with the following exception: for patients with Grade 2 AST, ALT, and/or alkaline phosphatase abnormalities at Baseline, only an increase to >8 × ULN lasting >48 hours will be considered a DLT   - Liver tests abnormalities meeting Hy’s law criteria   Hematologic toxicity   - - Any Grade 5 toxicity   - Any Grade 4 anemia   - Any ≥Grade 3 febrile neutropenia   - ≥Grade 4 neutropenia (absolute neutrophil count <500/µL) lasting >7 days   - ≥Grade 4 thrombocytopenia (<25,000/µL)   - ≥Grade 3 thrombocytopenia (<50,000/µL) lasting >7 days associated with clinically significant bleeding   TEAEs at least possibly related to SQZ-PBMC-HPV (alone or in combination) that result in permanent discontinuation or a delay >14 days of Cycle 2 Day 1 of scheduled SQZ‑PBMC‑HPV administration  Any other event that, in the judgement of the Investigator and Sponsor, is considered to be a DLT  In the event of an AE meeting the definition of a DLT but unrelated to SQZ‑PBMC-HPV, the findings will be discussed by the SSC (see Section 2.3). |
|  | **Additional Safety Evaluations:** Safety evaluations include incidence and severity of TEAEs and serious AEs as assessed by NCI CTCAE version 5.0; irAEs and signs of CRS; physical examination findings; ECOG PS; and clinically significant changes from Baseline in laboratory parameters, ECGs, LVEF, and vital signs.  **Preliminary Evidence of Antitumor Activity:** Preliminary evidence of antitumor activity of SQZ‑PBMC-HPV monotherapy and in combination with an immune checkpoint inhibitor(s) will be evaluated per RECIST 1.1 and iRECIST:  Progression-free survival  Disease control rate  Objective response rate  Stable disease lasting at least 12 weeks  Time to best overall response  Duration of response  Overall survival  **Dose Manufacturing Feasibility:** Dose manufacturing feasibility will be assessed based on individual patient batch yield, product failure prohibiting use, and any additional information from leukapheresis through SQZ-PBMC-HPV production that is deemed relevant to dose manufacturing feasibility.  **Immunogenic and Other Pharmacodynamic Evaluations:** Immunogenic assessments may include, but are not limited to, immunophenotyping, measurements of T cell biomarkers and cytokine production and circulating cell-free HPV16 DNA. In addition, changes in blood cytokines after treatment with SQZ-PBMC-HPV and pharmacodynamic assessments, including, but not limited to, determination of changes in blood samples, circulating blood cells, and cellular responses in tumor biopsies will be evaluated. Optional pharmacogenomic sequencing on tumor tissue and/or blood samples may also be conducted on samples collected for consenting patients. |
| Statistical Methods | No formal statistical hypothesis testing will be performed in Part 1. |

LIST OF ABBREVIATIONS AND DEFINITION OF TERMS

| **Abbreviation or Term** | **Definition** |
| --- | --- |
| AE | adverse event |
| AESI | adverse event of special interest |
| AIDS | acquired immunodeficiency syndrome |
| ALT | alanine aminotransferase |
| AST | aspartate aminotransferase |
| ASTCT | American Society for Transplantation and Cellular Therapy |
| β-hCG | Beta-human chorionic gonadotropin |
| BOR | best overall response |
| cfHPV DNA | circulating cell-free human papillomavirus strain 16 deoxyribonucleic acid |
| C1D1 | Cycle 1 Day 1 |
| C1D8 | Cycle 1 Day 8 |
| CFR | Code of Federal Regulations |
| cfDNA | circulating cell-free deoxyribonucleic acid |
| CNS | central nervous system |
| CPS | combined positive score |
| CR | complete response |
| CRF | case report form |
| CRO | contract research organization |
| CRS | cytokine release syndrome |
| CT | computed tomography |
| CTCAE | common terminology criteria for adverse events |
| CTLA-4 | cytotoxic T-lymphocyte-associated antigen 4 |
| DCR | disease control rate |
| DLT | dose-limiting toxicity |
| DNA | deoxyribonucleic acid |
| DoR | duration of response |
| DSMB | Data and Safety Monitoring Board |
| ECG | electrocardiogram |
| ECHO | echocardiogram |
| ECOG | Eastern Cooperative Oncology Group |
| eCRF | electronic case report form |
| EDC | electronic data capture |
| EODW3 | 3 weeks after the last administration of investigational product |
| EODW6 | 6 weeks after the last administration of investigational product |
| EMA | European Medicines Agency |
| Enrolled | A patient is considered to be enrolled in the study when he or she has signed the informed consent form. |
| ESR | expedited safety report |
| FDA | Food and Drug Administration |
| GCP | Good Clinical Practice |
| HBsAg | hepatitis B surface antigen |
| HBV | hepatitis B virus |
| HCV | hepatitis C virus |
| HIV | human immunodeficiency virus |
| HIV+ | human immunodeficiency virus positive |
| HLA | human leukocyte antigen |
| HLA-A*02 | human leukocyte antigen serotype within the HLA-A serotype group |
| HLA-A*02+ | human leukocyte antigen serotype within the HLA-A serotype group positive |
| HPV | human papillomavirus |
| HPV16 | human papillomavirus strain 16 |
| HPV16+ | human papillomavirus strain 16 positive |
| HPV18 | human papillomavirus strain 18 |
| IB | Investigator’s Brochure |
| IBC | Institutional Biosafety Committee |
| IC | tumor‑infiltrating immune cells |
| ICANS | immune effector cell-associated neurotoxicity syndrome |
| ICF | informed consent form |
| ICH | International Council for Harmonisation |
| iCPD | immune confirmed progression |
| iCR | immune complete response |
| IEC | Independent Ethics Committee |
| IFN | interferon |
| IFNɣ | interferon gamma |
| IL | interleukin |
| INR | international normalized ratio |
| iPR | immune partial response |
| irAE | immune-related adverse event |
| IRB | Institutional Review Board |
| iRECIST | modified RECIST criteria for incorporation into solid tumor studies of immunotherapeutics |
| IRR | infusion-related reaction |
| iSD | immune stable disease |
| iUPD | immune unconfirmed progressive disease |
| ITT | intent-to-treat |
| IV | intravenous(ly) |
| LTFU | long-term follow-up |
| LVEF | left ventricular ejection fraction |
| MAD | maximum administered dose |
| MedDRA | Medical Dictionary for Regulatory Activities |
| MRI | magnetic resonance imaging |
| MTD | maximum tolerated dose |
| MUGA | multi-gated acquisition scan |
| NCI | National Cancer Institute |
| NLNT | New Lesions-Non-Target |
| NLT | New Lesions-Target |
| OOS | out-of-specification |
| ORR | objective response rate |
| OS | overall survival |
| PBMC | peripheral blood mononuclear cell |
| PD | progressive disease |
| PD-1/PD-L1/PD-L2 | programmed death-1/ programmed death-ligand 1/ programmed death-ligand 2 |
| PFS | progression-free survival |
| PID | patient identification |
| PP | per-protocol |
| PR | partial response |
| pRB | retinoblastoma protein |
| PS | performance status |
| PT | prothrombin time |
| PTT | partial thromboplastin time |
| Q3W | every 3 weeks |
| QTcB | QTc corrected by Bazett’s formula |
| QTcF | QTc corrected by Fridericia’s formula |
| RECIST 1.1 | Response Evaluation Criteria for Solid Tumors version 1.1 |
| RNA | ribonucleic acid |
| RP2D | recommended Phase 2 dose |
| SAE | serious adverse event |
| SCCHN | squamous cell carcinoma of the head and neck |
| SD | stable disease |
| SLP | synthetic long peptide |
| SOP | standard operating procedure |
| SQZ-PBMC-HPV | the investigational drug product, consisting of autologous PBMCs presenting immunogenic epitopes of the E6 and E7 proteins of HPV16 |
| SSC | Study Safety Committee |
| SUSAR | suspected, unexpected serious adverse reaction |
| TEAE | treatment-emergent adverse event |
| TTMV | tumor-tissue modified virus |
| ULN | upper limit of normal |
| WBC | white blood cell |

SCHEDULE OF ASSESSMENTS

Details on procedures and timing of assessments for monotherapy and combination therapy are presented in **Table 1**.

**Table 1**. Schedule of Assessments: SQZ-PBMC-HPV Monotherapy, Dose Escalation.

| Assessments / Procedures | Screening | Leukapheresis^(b)^ (-1 Day) | Cycle 1^(a)^ | | | | Cycle 2 | | Cycles ≥3 | Post-treatment Follow-up | | |
| --- | --- | --- | --- | --- | --- | --- | --- | --- | --- | --- | --- | --- |
|  | Within 28 Days Prior to Leukapheresis |  | D1^® (^1st Dose) | D2 (Cohort 3 Only) | D8 (±2 Days) | D15^(d)^ (±2 Days) | D1 (±2 Days) | D8 (±2 Days) | D1 (±2 Days) | EODW3 (±3 Days) | EODW6^(e)^ (±3 Days) | LTFUP^(f)^ (±7 Days) |
| Informed ConsentP^(g)^ | X |  |  |  |  |  |  |  |  |  |  |  |
| Medical and Cancer HistoryP^(h)^ | X |  |  |  |  |  |  |  |  |  |  |  |
| Demography and Baseline Characteristics | X |  |  |  |  |  |  |  |  |  |  |  |
| Inclusion / Exclusion Criteria | X |  |  |  |  |  |  |  |  |  |  |  |
| Blood Sample for HLA-A*02 Status | X |  |  |  |  |  |  |  |  |  |  |  |
| Primary Tumor or Metastasis Biopsy^(i)^ | X |  |  |  |  |  |  | X^(Pj)^ |  |  |  |  |
|  |  | Unscheduled optional biopsies are requested at Cycle 5 Day 1 (predose) and if the patient progresses and discontinues treatment, if possible. Unscheduled biopsies may also be collected at additional time points to further characterize response and/or resistance mechanisms. | | | | | | | |  |  |  |
| Physical ExaminationP^(k)^ | X | X | X |  | X | X | X | X | X |  | X | X^f^ |
| ECOG Performance Status | X | X | X |  | X | X | X | X | X |  | X | X^f^ |
| Vital SignsP^(l)^ | X | X | X | X | X | X | X | X | X |  | X | X^f^ |
| 12-lead ECGP^(m)^ | X | X | X | X |  |  | X |  | X |  | X | X^f^ |
| ECHO / MUGA ScanP^(n)^ | X |  |  |  |  |  |  |  |  |  |  |  |
| Clinical Laboratory TestingP^(o)^ | X | X | X | X | X | X | X | X | X |  | X | X^f^ |
| Serum Pregnancy TestP^(p)^ | X |  | X |  |  |  | X |  | X |  | X |  |
| HBV, HCV, and HIV TestingP^(q)^ | X |  |  |  |  |  |  |  |  |  |  |  |
| Tumor Imaging | In all patients, tumor assessments will be performed at Screening (baseline), every 9 weeks (±7 days) for the first year, and every 12 weeks (±7 days) thereafter until disease progression, unacceptable toxicity, withdrawal of consent, death, or for 2 years from the date of the first administration of SQZ‑PBMC‑HPV, whichever occurs first. Patients who experience disease progression per RECIST 1.1 may continue dosing if considered in their best interest by the treating Investigator to allow for confirmation of disease progression, *i.e*. iCPD according to iRECIST (Seymour et al 2017). | | | | | | | | | | | |
| Blood sample for TTMV-HPV16 (cfD®^(r)^ | X |  |  |  |  |  |  |  |  |  |  |  |
| Blood Sample for cfDNA^(s)^ |  |  | X |  |  |  | X |  | X |  | X | X |
| Blood Sample for Pharmacodynamic Assessments^(s)^ | X |  |  |  | X | X | X | X | X |  | X | X |
| Leukapheresis^(b)(t)^ |  | X |  |  |  |  |  |  |  |  |  |  |
| Leukopak/Leukapheresis WBC Count^(u)^ |  | X |  |  |  |  |  |  |  |  |  |  |
| Pre-administration Medication AdministrationP^(v)^ |  |  | X | X |  |  | X |  | X |  |  |  |
| SQZ-PBMC-HPV AdministrationP^(w)^ |  |  | X | X |  |  | X |  | X |  |  |  |
| Blood Sample for Cytokine ProfileP^(x)^ |  |  | X | X | X | X | X | X | X |  | X |  |
| Adverse Events^(y)^ | **———————————————————————————————————————————————————————►** | | | | | | | | | | | X^f^ |
| Prior/Concomitant Medications^(z)^ | X | X | X | X | X | X | X | X | X | X | X | X^f^ |
| Survival Status |  |  |  |  |  |  |  |  |  |  |  | X |

Abbreviations: ASTCT=American Society for Transplantation and Cellular Therapy; cfDNA=circulating cell-free deoxyribonucleic acid; cfHPV DNA=circulating cell-free human papillomavirus strain 16 deoxyribonucleic acid; CRS=cytokine release syndrome; CTCAE=Common Terminology Criteria for Adverse Events; D=Day; ECG=electrocardiogram; ECHO=echocardiography; ECOG=Eastern Cooperative Oncology Group; EODW3=3 weeks after the last administration of investigational product; EODW6=6 weeks after the last administration of investigational product; HBsAg=hepatitis B surface antigen; HBV=hepatitis B virus; HCV=hepatitis C virus; HIV=human immunodeficiency virus; HLA-A*02=human leukocyte antigen serotype within the HLA-A serotype group; HLA-A*02+=human leukocyte antigen serotype within the HLA-A serotype group positive; HPV16=human papillomavirus 16; HPV16+=human papillomavirus 16 positive; iCPD=immune confirmed progression; iRECIST=modified RECIST criteria for incorporation into solid tumor studies of immunotherapeutics; LTFU=long-term follow-up; MUGA=multigated acquisition scan; NCI=National Cancer Institute; RECIST 1.1=Response Evaluation Criteria for Solid Tumors version 1.1; RNA=ribonucleic acid; TEAE=treatment-emergent adverse event; TTMV=tumor-tissue modified virus; WBC=white blood cell.

Note: Additional unscheduled safety or efficacy assessments may be performed at any time as clinically indicated to determine the relevance of specific findings and/or the duration of events.

1. Patients will receive administration of SQZ‑PBMC-HPV at 3-week intervals until treatment discontinuation criteria are met or until the SQZ-PBMC-HPV supply is exhausted or for a maximum of 1 year, whatever comes first. Patients who experience disease progression per RECIST 1.1 may continue dosing if considered in their best interest by the treating Investigator to allow for confirmation of disease progression, *i*.*e*. iCPD according to iRECIST.
2. Leukapheresis should typically occur 8 to 14 days prior to the first administration of SQZ-PBMC-HPV. Scheduling of the first administration of SQZ‑PBMC‑HPV will take into account site location and shipping logistics.
3. On Cycle 1 Day 1, a physical examination, ECOG, vital signs, clinical laboratory testing, and a blood sample for cytokines should be completed prior to administration of SQZ-PBMC-HPV. These assessments may also occur the day prior to SQZ‑PBMC‑HPV administration.
4. There will be no Cycle 1 Day 15 visit in the Expansion Phase.
5. EODW6 will serve as the Safety Follow-up Visit.
6. Following completion of the EODW6 visit, patients will enter the LTFU period. Patients experiencing ongoing TEAEs related to study treatment should continue to be followed as clinically indicated until TEAEs resolve to Grade 1 or are deemed irreversible. Patients who discontinue study treatment for reasons other than progressive disease will continue to undergo tumor assessment according to the protocol schedule until progressive disease is documented. Once treatment-related adverse events have resolved and progressive disease is documented, patients will be followed for survival only. Patients will be followed for survival for up to 2 years from the date of the first administration of SQZ-PBMC-HPV. Contact for adverse events and survival follow-up should occur every 3 months at a minimum and can be conducted via telephone.
7. A study informed consent form will be signed prior to any Screening assessments that are not considered standard of care.
8. Screening medical and cancer history to include confirmation, if available, of HLA-A*02+ and HPV16+ status by pathology report via a certified laboratory.
9. Fresh tumor biopsy taken at Screening for HPV16+ confirmation (if applicable) and pharmacodynamic assessments (required) should be from the primary tumor or metastasis. The required tumor biopsy for pharmacodynamic assessments may be collected after confirming HPV16+ status locally or via TTMV-HPV16 (cfDNA) whole blood. The same lesion should be biopsied at screening/baseline and C2D8; if that is not feasible, a similar mass in the same anatomical location should be biopsied. Pharmacogenomic testing may be completed on samples collected from consenting patients.
10. All patients are required to undergo a repeat tumor biopsy at Cycle 2 Day 8. Biopsies should be from the same primary tumor or metastasis biopsied at Screening. If that location is not feasible a similar mass in the same anatomical location should be biopsied.
11. Complete physical examination includes height (Screening only), weight, neurological examination, and evaluation of signs and symptoms. The weight recorded within 24 hours must be used for leukapheresis.
12. Vital signs include blood pressure, respiratory rate, heart rate, and temperature. On days of SQZ-PBMC-HPV administration, evaluate vitals prior to SQZ‑PBMC-HPV administration within 5 minutes before any SQZ-PBMC-HPV syringe administration and 30 minutes, 1, 2, and 4 hours post SQZ-PBMC-HPV syringe administration. On days without an SQZ-PBMC-HPV administration, collect vital signs during visit. A window of ±10 minutes is permissible for vital sign evaluation.
13. On days when SQZ-PBMC-HPV is administered, an ECG should be performed at the following time points: 30 minutes prior to administration (+ 10 mins), 30 minutes after administration (+ 10 mins), and 2 hours and 4 hours post administration (a window of ±30 minutes is acceptable for the 2- and 4‑hour post administration ECG). On days when no SQZ‑PBMC‑HPV is administered, an ECG can be performed any time during visit. Please have the patient in a resting position for at least 10 minutes prior to ECG.
14. ECHO/MUGA at Screening and as clinically indicated.
15. Clinical laboratory testing will be performed locally; refer to Table 1 for a complete list of assessments, the days they are required to be collected, and when results are required to be available.
16. In women of childbearing potential, a serum pregnancy test will be performed at Screening. Serum or urine pregnancy tests will be performed on Day 1 of each subsequent cycle and EODW6.
17. If positive for anti-HCV, then HCV RNA testing will be performed. Patients with active HBV (*i.e*. positive for HBsAg) or HCV (*i.e*. positive HCV RNA results) infections are not eligible. Screen all patients for HIV; test results for patients who screen positive for HIV require a discussion with the Sponsor prior to determination of eligibility for study.
18. If the screening TTMV-HPV16 (cfDNA) sample is not collected for testing, the quality/quantity is insufficient for testing, the result is negative, or the investigator otherwise considers appropriate, proceed to primary tumor or metastasis biopsy for confirmation of HPV16+ status after input from the sponsor.
19. When the cfDNA or pharmacodynamic assessment sample collection occurs on a dosing day, ensure that the blood sample(s) for assessments is collected before SQZ-PBMC-HPV administration. Pharmacogenomic testing may be completed on samples collected from consenting patients.
20. Results of the physical examination, vital signs, ECOG score, and ECGs are required to be available prior to leukapheresis. Laboratory results required to be available prior to leukapheresis include hematology and coagulation profile.
21. If possible, a blood sample should be collected for WBC count during leukapheresis or at the end of leukapheresis from the leukopak. The results should be processed as soon as possible and provided to the Sponsor in real time. Leukopak cell concentrate from leukapheresis will be shipped for manufacture of SQZ-PBMC-HPV.
22. All patients should be pre-medicated with 650 mg acetaminophen orally and 25 mg diphenhydramine intravenously or orally (or another H1 antagonist at equipotent dose) approximately 30 to 60 minutes prior to initiation of each administration of SQZ-PBMC-HPV.
23. All patients in the study will be observed for at least 4 hours after each administration of SQZ‑PBMC-HPV. The first 2 patients in all cohorts will undergo a minimum of 23 hours of observation after the first administration of SQZ-PBMC-HPV and at least 4 hours after each subsequent administration. In Cohort 3, the second administration on Cycle 1 Day 2 should be given during the ≥23-hour observation. Patients will be observed for a minimum of 4 hours after the second priming administration. The minimum interval between the 2 administrations should be 16 hours.
24. For all patients on Day 1 of each cycle when SQZ‑PBMC‑HPV is administered, a blood sample for cytokine profile should be taken 30 minutes before and at 1, 2, and 4 hours post administration. For the first 2 patients in Cohorts 1, 2, and 3 (who will undergo a minimum of 23 hours of observation after the first administration of SQZ-PBMC-HPV), blood samples for cytokines will be collected at 30 minutes before and at 1, 2, 4, 6, 8 to 10 (1 draw during this window), and 24 hours post administration. On Cycle 1 Day 2 (Cohort 3), a blood sample for cytokine profile should be taken 30 minutes before and 4 hours post administration. The 24-hour-post-administration sample might overlap with either the ’30-minutes-before’ or the ‘4-hours-post-administration’ sample. In such a situation, a single sample for each time point is sufficient. For all other visits (including unscheduled visits), a single blood sample for cytokines should be taken. A window of ±10 minutes is permissible for blood sample collection for cytokines. Patients with Grade 2, 3, or 4 CRS will have additional cytokine plasma levels performed during Grade 2, 3, or 4 CRS events. Blood collections should be obtained at time of diagnosis of a CRS, at time of an increase in severity (*e*.*g*. when a Grade 2 CRS progresses to a Grade 3 CRS), onset of neurological symptoms, and at time of discharge or resolution.
25. All patients will be monitored for safety, including CRS and neurotoxicity. Grading of CRS and neurotoxicity will use the ASTCT Consensus Grading. Adverse events will be evaluated as per NCI CTCAE version 5.0. Treatment-emergent adverse events and any serious adverse events that are ongoing at the time of the latest visit should continue to be followed until resolution or until return to Baseline as determined at the time of the pre-first infusion visit.
26. At each study visit, patients will be asked whether they have taken any medication, received a transfusion, or received treatment other than the investigational product after the first dose of investigational product.
27. Other Protocol Particulars
    1. Definition of End of the Clinical Study

Following completion of study treatment, *i*.*e.* permanent discontinuation of SQZ-PBMC-HPV and/or the immune checkpoint inhibitor(s), patients will undergo safety follow up until treatment‑related AEs resolve. The patient will then enter the long-term follow-up phase of the study. The end of the clinical study (*i*.*e.* completion of the study at all participating study sites) is defined as the date that the last patient in the study discontinues long-term follow up due to death, withdrawal of consent, or are deemed by the investigator to be lost to follow up.

- 1. Study Stopping Rules

The clinical study may be stopped at any time and dosing may be halted temporarily to investigate safety and tolerability or secondary endpoint before the entire study is terminated.

If the Investigator, Medical Monitor, or Sponsor becomes aware of conditions or events that suggest a possible hazard to patients, then the clinical study may be terminated by the Sponsor after appropriate consultation among the involved parties. The clinical study may be terminated at the Sponsor’s discretion also in the absence of such a finding.

Should the study be terminated and/or a study site closed for any reason, all documentation pertaining to the study and study drug must be returned to the Sponsor. Any actions of the contract research organization (CRO) required for assessing or maintaining patient safety will continue as required, despite termination of the study by the Sponsor.

- 1. Study Safety Committee and Data Safety Monitoring Board

This study will employ 2 oversight committees: (1) the SSC and (2) the DSMB.

The SSC is comprised of the Principal Investigators of all active sites, the Medical Monitor, and SQZ Biotechnologies representatives. The SSC will be updated on safety throughout the study. It is recognized that the SSC interactions will be in part driven by enrollment in the study and safety events throughout the study. The SSC will review available safety data and confirm any DLT, if applicable.

The following decisions are made by the SSC:

- Review of safety observations in escalation cohorts to determine initiation of subsequent cohorts (or continue enrollment in open cohorts for further assessment) and magnitude of dose escalation or de-escalation (selection of lower dose level that could be single or double prime), if applicable. Per the SSC charter, the DSMB may be consulted.
- For Grade 3 IRR, the SSC makes a recommendation if changes to premedication or a modification of the administration rate should be applicable to all subsequent patients enrolled in the study.
- Review of safety data to complete safety assessment and decision to initiate enrollment in the Combination Safety Phase (recommendation to DSMB).
- Review of safety data to complete safety assessment and decision to initiate enrollment in the Monotherapy Expansion Phase (recommendation to DSMB).

The SSC will also participate in the selection of the RP2D regimen for monotherapy and combination therapy by providing recommendations to the DSMB.

A charter for the SSC has been established.

To ensure patient safety during the dose expansion part of the study, an independent DSMB will be established prior to opening the combination therapy cohorts. The DSMB will convene to define the RP2D for the combination phase and periodically, if needed, to monitor the safety data of the patients. The DSMB will decide, by consensus, on continuation, modification, or suspension of the trial or of a particular combination therapy cohort. Meeting frequency and details regarding the data to be reviewed are described in the DSMB Charter. The DSMB and the Sponsor may modify the frequency of meetings, as deemed appropriate, during the course of the trial.

The SSC may decide to escalate decisions regarding continuation, modification, or suspension of the trial or of a particular combination therapy cohort. In such cases, the DSMB will render decisions by consensus.

Details regarding the remit of both the SSC and DSMB are outlined in **Table 2**.

| **Table 2.** Responsibilities of the SSC and DSMB | | |
| --- | --- | --- |
| Decision/Remit | SSC^b^ | DSMB |
| Part 1, Monotherapy: |  |  |
| - Determine initiation of subsequent cohorts (or continue enrollment in open cohorts for further assessment) | ✓ |  |
| - Determine magnitude of dose escalation or de-escalation (selection of lower dose level that could be single or double prime) | ✓ |  |
| - RP2D determination (makes recommendation to DSMB) | ✓ |  |
| - Decision to transition from Part 1 of the Study to Part 2 and Part 3^a^ | ✓ | ✓ |
| For Grade 3 IRR, makes a recommendation if changes to premedication or a modification of the administration rate should be applicable to all subsequent patients enrolled in the study. | ✓ |  |
| Decision to de-escalate dose | ✓ |  |
| Decision to employ single prime or double prime SQZ-PBMC-HPV as part of the RP2D | ✓ |  |
| Transition to Cohort 7 (SQZ-PBMC-HPV + nivolumab + ipilimumab) | ✓ |  |
| Decision to dose de-escalate (Part 2, Combination Safety Phase and Part 3, Monotherapy Dose Expansion) | ✓ |  |
| Ad hoc review if DLT rate >33% (Part 2 and 3) | ✓ |  |
| Transition to Stage 2 (min/max or Simon 2-stage) in Part 3 – Monotherapy Dose Expansion | ✓ |  |

Abbreviations: DLT=dose-limiting toxicity; DSMB=Data and Safety Monitoring Board; IRR=infusion-related reaction; RP2D=recommended phase 2 dose; SSC=Study Safety Committee.

^a^ The SSC will make the RP2D recommendation, which will be ratified by the DSMB.

^b^ Decisions may be escalated to DSMB for adjudication.

- 1. Stopping Criteria for a Cohort and Stopping of Dose Escalation or Progression to Cohort and Termination of Study

The modified 3+3 rule define the ultimate decision to declare a cohort as safe. The minimum number of patients needed to confirm a cohort as safe is 3 patients with 0 DLTs, which can be increased up to 12 patients to confirm that a cohort is safe (*i*.*e.* <33% of patients with DLT; for instance, 6 patients with <2 DLTs, 9 patients with <3 DLTs, or 12 patients with <4 DLTs, whichever confirms the safety of the cohort). If none of the cohorts indicate that the MTD has been reached, additional cell dose levels or regimens may be tested. In the event of AEs covered by the definition of a DLT but unrelated to SQZ-PBMC-HPV, the findings will be discussed by the SSC.

An AE that meets the definition of a DLT and occurring outside the DLT window will not be counted as a DLT but instead will be considered in the overall safety assessment of a given cohort and the selection of an RP2D regimen.

The cohort stopping rule is the occurrence of >3 DLTs in up to 12 patients (≥33%) receiving investigational product within the same dose cohort. If the stopping rule is triggered, the SSC may make 1 of the following recommendations:

- Declare the prior tolerated dose level as the MTD.
- Declare a dose level the MAD level without observation of a DLT. Thus, the RP2D would not be the MTD.
- Recommend testing of an intermediate dose level.
- Recommend protocol amendment to increase patient safety.
- Discontinue enrollment and/or the study.
- Following review by the SSC, dosing of patients may be stopped in the interest of patient safety based on these general safety criteria:
- Any SAE that is considered potentially life-threatening and is assessed by the Medical Monitor as related to investigational product.
- Any other clinically significant change that indicates to the Investigator or Sponsor a major tolerability concern.
  1. Infusion-Related Reaction

Guidelines regarding dose interruptions and management of IRRs are described in Table 3.

**Table 3.** Management of Infusion-Related Reactions

| Grade | Management |
| --- | --- |
| 1 | - Infusion interruption not indicated - Monitor until recovery from symptoms - Consider modification of premedication for subsequent infusions as per institutional guidelines |
| Mild transient reaction; infusion interruption not indicated; intervention not indicated |  |
| 2 | - Stop infusion and halt any thawing process until resolution to ≤Grade 1 - Begin infusion of normal saline - Provide symptomatic treatment with antipyretics, analgesics, histamine blockers (H1 and H2) and bronchodilators as needed - Provide symptomatic corticosteroids when other symptomatic treatment does not lead to symptom improvement - Once resolves to ≤Grade 1, re-initiate administration at 50% of original rate and titrate to tolerance - Provide intensified premedication for subsequent infusions as per institutional guidelines - For subsequent infusions, start infusion at 50% of rate at which symptoms occurred and titrate to tolerance |
| Therapy or infusion interruption indicated but responds promptly to symptomatic treatment (*e*.*g*. antihistamines, nonsteroidal anti-inflammatories, narcotics, intravenous fluids); prophylactic medications indicated for ≤24 hours |  |
| 3 |  |
| Prolonged (*e*.*g*. not rapidly responsive to symptomatic medication and/or brief interruption of infusion); recurrence of symptoms following initial improvement; hospitalization indicated for clinical sequelae |  |
| 4 | - Permanently stop infusion, and permanently discontinue offending agent(s) - Manage severe to life-threatening infusion-related reaction as per institutional standards (*e*.*g*. epinephrine, histamine blockers [H1 and H2], corticosteroids, bronchodilators, oxygen, fluids) - Monitor patient carefully for at least 24 hours |
| Life-threatening consequences; urgent intervention indicated |  |

Source: (3, 4)

All patients should be counseled regarding recognizing signs and symptoms of immune-related reactions and advised to contact their treating physician should they occur.

- 1. Cytokine Release Syndrome

Occurrence of a treatment‑emergent Grade 3/4 CRS event or neurotoxicity that does not resolve to <Grade 2 within 24 hours would trigger an ad hoc safety meeting to address this AE. The totality of safety events observed will be reviewed (including all CRS events) and a decision will be made if this event requires initiation of staggered enrollment of patients. The procedure of staggered enrollment requires that the first treatment of all subsequent patients is staggered by 1 week and all patients will return to the clinic for assessment 48 to 72 hours following the first administration. In patients with CRS, the following laboratory parameters should be obtained: cytokine plasma levels, coagulation (PT, PTT/INR, D-dimer, fibrinogen, and von Willebrand factor), liver function tests (AST, ALT, and total bilirubin), and comprehensive metabolic panel, including creatinine, complete blood count, C‑reactive protein, and ferritin. All affected patients will have safety assessments including cytokine plasma levels tested during the unscheduled visits based on the Schedule of Assessments. Patients with Grade 2, 3, or 4 CRS will have additional cytokine plasma levels performed during Grade 2, 3, or 4 CRS events.

According to the NCI dictionary of cancer terms CRS is caused by a large, rapid release of cytokines into the blood from immune cells affected by the immunotherapy (5). Cytokines are immune substances that have many different actions in the body. Signs and symptoms of CRS include fever, nausea, headache, rash, rapid heartbeat, low blood pressure, and trouble breathing. Most patients experience a mild reaction, but the reaction may be severe or life-threatening in some patients.

The guidelines and recommendations for grading and managing CRS are described in Table 4. The Medical Monitor/Sponsor should be contacted for any Grade ≥2 CRS; CRS of Grade 2 or higher are AESIs and must be reported to the Sponsor within 24 hours of awareness.

**Table 4**. Management of Cytokine Release Syndrome

| GradeP^a^ | ManagementP^b^ | |
| --- | --- | --- |
| **1** | - Stop infusion until resolution to Baseline (if the CRS is occurring during the actual infusion) - Assess for infection - Provide symptomatic treatment with antipyretics, analgesics, histamine blockers (H1 and H2) and corticosteroids as needed | - Monitor fluid balance - Once resolves to Baseline, re-initiate infusion at 50% of original rate - Consider premedication for subsequent infusions as per institutional guidelines |
| Fever (≥38°C) not attributable to any other cause  Hypotension - None  Hypoxia - None |  |  |
| **2** | - Stop infusion until resolution to Baseline (if the CRS is occurring during the actual infusion) - Assess for infection - Provide symptomatic treatment with antipyretics, analgesics, histamine blockers (H1 and H2) and corticosteroids as needed - Treat hypotension with IV fluid for blood pressure support - Administer oxygen as needed | - Monitor cardiac and other organs function closely - Consider tocilizumab - Once resolves to Baseline, re-initiate infusion at 50% of original rate - Provide premedication for subsequent infusions as per institutional guidelines - For subsequent infusions, start infusion at 50% of rate at which symptoms occurred and titrate to tolerance |
| Fever (≥38.0°C) not attributable to any other cause  **with**  Hypotension - not requiring vasopressors  **and/or**  Hypoxia - requiring the use of oxygen delivered by low-flow nasal cannula (≤6 L/minute) or blow-by |  |  |
| **3** | - If the CRS is occurring during the actual infusion, permanently stop administration - Assess for infection - Provide symptomatic treatment with antipyretics, analgesics, histamine blockers (H1 and H2) and corticosteroids as needed - Treat hypotension with IV fluid for blood pressure support and/or pressor - Administer oxygen as needed - Monitor cardiac and other organs function closely - Administer tocilizumab as per tocilizumab (ActemraP^®^P) prescribing information for treatment of CRS, ±corticosteroids | |
| Fever (≥38.0°C) not attributable to any other cause  **with**  Hypotension - requiring a vasopressor with or without vasopressin  **and/or**  Hypoxia - requiring high-flow nasal cannula (>6 L/minute), facemask, nonrebreather mask, or venturi mask |  |  |
| **4** |  |  |
| Fever (≥38.0°C) not attributable to any other cause  **with**  Hypotension - requiring multiple vasopressors (excluding vasopressin)  **and/or**  Hypoxia - requiring positive pressure (*e*.*g*. CPAP, bilevel positive airway pressure, intubation, mechanical ventilation). |  |  |

Abbreviations: CPAP=continuous positive airway pressure; CRS=cytokine release syndrome; IV=intravenous.

Note: Cytokine release syndrome rarely occurs during the infusion. In most cases, onset occurs hours to days after dosing.

^a^ (6)

^b^ (4, 7, 8)

- 1. DISCONTINUATION
     1. Patient Withdrawal and Replacement

In cohorts with ongoing DLT assessment, patients who are not eligible for DLT assessment due to a missed dose of SQZ-PBMC-HPV for reasons other than safety during the Combination Safety Assessment must be replaced.

Patients will be removed from the investigational product and/or the checkpoint inhibitor when any of the criteria listed below occurs. The primary reason for study treatment discontinuation must be documented.

A patient must be discontinued from study treatment (but will continue to be monitored in the post‑treatment follow-up portion of the study) for any of the following reasons:

- Patient or legal representative (such as a parent or legal guardian) withdraws investigational product consent for treatment.
- Documented disease progression. Patients who experience disease progression per RECIST 1.1 may continue dosing if considered in their best interest by the treating Investigator to allow for confirmation of disease progression, *i*.*e.* iCPD according to iRECIST (1).
- Available autologous drug product is exhausted (monotherapy cohorts).
- Discontinuation is considered necessary by the Investigator and/or Sponsor due to occurrence of an exclusion criterion or AE that is clinically relevant and affects the patient’s safety. In case of a discontinuation due to an AE, the patient must be followed until resolution, the condition stabilizes, or the Investigator and Sponsor agree that follow up is not required, unless the patient withdraws consent.
- Patients experiencing a DLT that is not an IRR will be discontinued from the study. If, in the opinion of the Investigator and the Sponsor, it is in the patient’s best interest to continue treatment with investigational product, then the subsequent treatment will be determined by the Investigator in consultation with the Sponsor. For IRRs, the premedication or rate of administration may be adjusted to enable the patient to remain on study.
- Need for >2 dose delays due to the same toxicity as per the dose delay guidelines.
- Noncompliance with investigational product or procedure that is deemed by the Investigator or the Sponsor to compromise the patient’s safety or study integrity.
- Lost to follow-up.
- Patient becomes pregnant.

If a patient withdraws prematurely from the study after dosing is completed, then the patient should return to the site within 15 to 45 days of treatment discontinuation for a Safety Follow-up visit (EODW6 assessment visit).

Following completion of the EODW6 visit, patients will enter the long-term follow up (LTFU) period. Patients experiencing ongoing TEAEs related to study treatment should continue to be followed as clinically indicated until TEAEs resolve to Grade 1 or are deemed irreversible. Patients who discontinue study treatment for reasons other than PD will continue to undergo tumor assessment according to the protocol schedule until PD is documented. Once treatment-related AEs have resolved and PD is documented, patients will be followed for survival only. Patients will be followed for survival for up to 2 years from the date of the first administration of SQZ‑PBMC‑HPV. Contact for AEs and survival follow up should occur every 3 months at a minimum and can be conducted via telephone.

1. Product Testing and Histochemistry Methods
   1. SQZ-PBMC-HPV Release Testing

| **Table 5.** Overview of Analytical Test Methods | |
| --- | --- |
| **Parameter** | **Test Method^a^** |
| - Appearance | - Visual |
| - Identity (Composition and Frequency) | - Flow cytometer |
| - Dose – Total Cell Count | - Cell counter |
| - Dose – Viability % | - Cell counter |
| - pH | - USP<791> |
| - Endotoxin | - USP<85>, Endosafe PTS^™^, kinetic chromogenic LAL assay |
| - Mycoplasma | - USP <1223>/ 21 CFR 610.9, MycoSEQ^™^ real-time PCR |
| - Sterility | - 21 CFR 610.12, Milliflex^®^ Rapid, ATP bioluminescence |
| - ^a^Release tests were performed after thawing of the SQZ-PBMC-HPV drug product. | |

**Appearance**: Thawed SQZ-PBMC-HPV is evaluated visually for color against a white background, homogeneity of the cell suspension against a black background, and foreign particulates against both backgrounds after gentle swirling of the contents.

**Identity (Composition and Frequency)**: Thawed SQZ-PBMC-HPV was stained with DRAQ5 (detects all nucleated cells), LIVE/DEAD™ Fixable Aqua (distinguishes live vs. dead cells by limited staining at the surface of live cells), and anti-human CD45-FITC conjugated antibody. Stained SQZ-PBMC-HPV was then analyzed by flow cytometry, gating to count live nucleated cells (DRAQ5+, low LIVE/DEAD™ Fixable Aqua), and the CD45+ proportion within the live nucleated cells.

**Dose (Total Cell Count)**: An image-cytometry based cell counter was used to determine the total number of cells/mL in thawed SQZ-PBMC-HPV. The latter was appropriately diluted and stained with acridine orange, which stains all nucleated cells, then read via image-cytometry. The total cell count was obtained by counting acridine orange positive cells within the imaging frame, then scaling by the relevant dilution factors.

**Dose (Viability %)**: An image-cytometry based cell counter was used to determine the % viability in thawed SQZ-PBMC-HPV, in a manner analogous to the method for the total cell count. An appropriately diluted aliquot of cells was stained with both acridine orange (staining all nucleated cells) and DAPI (4′,6-diamidino-2-phenylindole, staining only dead cells), followed by reading via image cytometry. A total cell count was obtained by counting acridine orange positive cells within the imaging frame; the count of dead cells derived from the DAPI positive cells within the imaging frame.

Viability %=100 × (Total cells – Dead cells)/Total Cells

**pH**: The pH of thawed SQZ-PBMC-HPV was determined by a conventional pH meter after calibration of the pH electrode.

**Endotoxin**: Endotoxin levels were measured by a chromogenic assay using an Endosafe PTS™ instrument and USP/BET compliant Endosafe® disposable cartridges.

**Mycoplasma**: The presence or absence of mycoplasma DNA was determined by the MycoSEQ™ Mycoplasma Detection Assay, which is a qPCR test using an array of primers specific to over 90 mycoplasm species.

**Sterility**: The presence or absence of microorganisms was determined using the Milliflex® Rapid System.

- 1. BIOPSY ANALYSES

IHC, IF and RNA ISH analyses were performed using both Ventana and Leica autostainers. The assays applied the antibodies or probes shown in Supplemental **Table** **6**. Brightfield and fluorescent images were digitized and analyzed by Digital image analysis (HALO or Visiopharm) or Pathologist scoring.

| **Table 6.** Staining antibodies and *in* *situ* hybridization probes used in the analysis of patient biopsies. | | | |
| --- | --- | --- | --- |
| **Target** | **Target**  **Sequence** | **Antibody Clone or**  **ISH Probe** | **Antibody**  **Isotype** |
| CD8 (CD8/PanCK) | Human CD8α,  C-terminus | SP239 | Rabbit  Monoclonal  IgG |
| PanCK | Human Cytokeratins  (Type I&II) | AE1/AE3/PCK26 | Mouse  Monoclonal  IgG1 Cocktail |
| CD8 (CD8/GZMB/FoxP3) | Human CD8α,  C-terminus of Alpha chain | C8/144B | Mouse  Monoclonal  IgG1κ |
| Granzyme B | Human  Granzyme B | EPR8260 | Rabbit  Monoclonal  IgG |
| FoxP3 | Human FoxP3  aa 105-236 | 236A/E7 | Mouse  Monoclonal  IgG1κ |
| PD-L1 | Human PD-L1  (B7-H1 or CD274)  aa 284–290 | SP263 | Rabbit  Monoclonal  IgG |
| MHC-I | Human HLA A  aa 50-150 | EP1395Y | Rabbit  Monoclonal  IgG |
| HPV16 E6 | HPV16-E6  nt 2 - 476 | RNAscope® 2.5 LS Probe-V-HPV16-E6 (Cat No 450598) | NA |
| HPV16 E7 | HPV16-E7  nt 563 - 846 | RNAscope® 2.5 LS Probe-V-HPV16-E7 (Cat No 463468) | NA |

1. REFERENCES

1. Seymour L, Bogaerts J, Perrone A, Ford R, Schwartz LH, Mandrekar S*, et al.* iRECIST: guidelines for response criteria for use in trials testing immunotherapeutics. *Lancet Oncol* **2017**;18:e143-e52.

2. Eisenhauer EA, Therasse P, Bogaerts J, Schwartz LH, Sargent D, Ford R*, et al.* New response evaluation criteria in solid tumours: revised RECIST guideline (version 1.1). *Eur J Cancer* **2009**;45:228-47.

3. Rosello S, Blasco I, Garcia Fabregat L, Cervantes A, Jordan K, and Committee EG. Management of infusion reactions to systemic anticancer therapy: ESMO Clinical Practice Guidelines. *Ann Oncol* **2017**;28:iv100-iv18.

4. Rosello S, Blasco I, Garcia Fabregat L, Cervantes A, Jordan K, and Committee EG. Management of infusion reactions to systemic anticancer therapy: ESMO Clinical Practice Guidelines. *Ann Oncol* **2018**;29:iv260.

5. NCI Dictionary of Cancer Terms. Available from: <https://www.cancer.gov/publications/dictionaries/cancer-terms/def/cure>. Accessed: 2019.

6. Lee DW, Santomasso BD, Locke FL, Ghobadi A, Turtle CJ, Brudno JN*, et al.* ASTCT Consensus Grading for Cytokine Release Syndrome and Neurologic Toxicity Associated with Immune Effector Cells. *Biol Blood Marrow Transplant* **2019**;25:625-38.

7. Lee DW, Gardner R, Porter DL, Louis CU, Ahmed N, Jensen M*, et al.* Current concepts in the diagnosis and management of cytokine release syndrome. *Blood* **2014**;124:188-95.

8. Karon A. How to manage cytokine release syndrome. Available from: <https://www.mdedge.com/hematologynews/article/155400/cellular-therapy/how-manage-cytokine-release-syndrome>. Updated: January 3, 2018.
