# Supplementary material for: Phase 1 study to determine the safety and dosing of autologous PBMCs modified to present HPV16 antigens (SQZ-PBMC-HPV) in HLA-A*02+ patients with HPV16+ solid tumors
Source: Invest New Drugs. Author manuscript; Available in PMC 2024 Apr 1. (PMC10140074; doi:10.1007/s10637-023-01342-x)
Supplement: 2 [file NIHMS1895206-supplement-2.docx]

Supplemental tables and figures accompanying:

Phase 1 study to determine safety, tolerability, and recommended dose of autologous PBMCs modified for antigen presentation targeting HPV16 (SQZ-PBMC-HPV) in HLA-A*02+ patients with HPV16+ Solid Tumors

**Table S1**. Disposition of all subjects from escalating dose evaluation of SQZ-PBMC-HPV.

**Table S2**. All treatment emergent adverse events from escalating dose evaluation of SQZ-PBMC-HPV.

**Table S3.** All related (TEAEs) from escalating dose evaluation of SQZ-PBMC-HPV, with breakout by grade.

**Table S4.** Summary of best overall response per RECIST1.1 and percent change in CD8+, E6, and E7

**Figure S1.** Flow chart with disposition of all subjects from escalating dose evaluation of SQZ-PBMC-HPV monotherapy.

**Figure S2.** Serum Cytokine Levels: IL-2, IFNγ, TNFɑ.

**Figure S3.** Swim lane plot of response assessment and time on treatment

**Figure S4.** Histology results for FoxP3+ cell density in tumor tissue, utilizing paired biopsies from subjects in escalating dose evaluation of SQZ-PBMC-HPV.

**Figure S5.** Histology results for CD8+/GZMB+ cell density in tumor tissue, utilizing paired biopsies from subjects in escalating dose evaluation of SQZ-PBMC-HPV.

**Figure S6.** Transcript levels of HPV16 E6 in paired biopsies from subjects in escalating dose evaluation of SQZ-PBMC-HPV.

**Figure S7.** Transcript levels of HPV16 E7 by in situ hybridization, in paired biopsies from subjects in escalating dose evaluation of SQZ-PBMC-HPV.

**Figure S8.** Percentage of tumor cells with PD-L1 membrane staining, measured histologically in paired biopsies from subjects in escalating dose evaluation of SQZ-PBMC-HPV.

**Figure S9.** MHC-I expression in paired biopsies from subjects in escalating dose evaluation of SQZ-PBMC-HPV.

**Table S1.** Disposition of all subjects from escalating dose evaluation of SQZ-PBMC-HPV. Entries give the number of subjects and the corresponding percentage of the number of patients in the respective cohort, or the study in its entirety.

|  | **Low Dose -**  **Single Prime** 0.5x10^6^ live cells/kg (n=3) | **Intermediate Dose - Single Prime** 2.5x10^6^ live cells/kg (n=5) | **Intermediate Dose - Double Prime** 2.5x10^6^ live cells/kg DP (n=4) | **High Dose –**  **Double Prime** 5x10^6^ live cells/kg DP (n=6) | **Total (N=18)** |
| --- | --- | --- | --- | --- | --- |
| **Received Leukapheresis** | 3 | 5 | 4 | 7 | 19 |
| **Dosed** | 3 (100.0) | 5 (100.0) | 4 (100.0) | 6 (100.0) | 18 (100.0) |
|  | | | | | |
| **Active** | 0 (0.0) | 0 (0.0) | 0 (0.0) | 2 (33.3) | 2 (11.1) |
|  | | | | | |
| **No Longer on Treatment** | **3 (100.0)** | **5 (100.0)** | **4 (100.0)** | **4 (66.7)** | **16 (88.9)** |
| Completed Planned Treatment/Exhausted Supply | 2 (66.7) | 1 (20.0) | 0 (0.0) | 2 (33.3) | 5 (27.8) |
| Discontinued prior to Treatment Completion | 1 (33.3) | 4 (80.0) | 4 (100.0) | 2 (33.3) | 11 (61.1) |
| Withdrawal of Consent from Treatment Only | 0 (0.0) | 1 (20.0) | 0 (0.0) | 0 (0.0) | 1 (5.6) |
| Progressive Disease | 1 (33.3) | 3 (60.0) | 3 (75.0) | 2 (33.3) | 9 (50.0) |
| Death | 0 (0.0) | 0 (0.0) | 1 (25.0) | 0 (0.0) | 1 (5.6) |
|  | | | | | |
| **Off Treatment but Still on Study** | 2 (66.7) | 1 (20.0) | 1 (25.0) | 4 (66.7) | 8 (44.4) |
|  |  |  |  |  |  |
| **Off Treatment, Off Study** | **1 (33.3)** | **4 (80.0)** | **3 (75.0)** | **0 (0.0)** | **8 (44.4)** |
| Death | 1 (33.3) | 3 (60.0) | 3 (75.0) | 0 (0.0) | 7 (38.9) |
| Other | 0 (0.0) | 1 (20.0) | 0 (0.0) | 0 (0.0) | 1 (5.6) |
|  | | | | | |
| **Deaths** | 1 (33.3) | 4 (80.0) | 3 (75.0) | 0 (0.0) | 8 (44.4) |
| **Deaths within 30 days of Last Dose** | 0 (0.0) | 0 (0.0) | 2 (50.0) | 0 (0.0) | 2 (11.1) |

**Supplemental Table 2.** All treatment emergent adverse events from escalating dose evaluation of SQZ-PBMC-HPV. Entries give the number of subjects and the corresponding percentage of the number of patients in the respective cohort, or the study in its entirety.

| **MedDRA**  **Preferred Term** | | **Low Dose - Single Prime** 0.5x10^6^ live cells/kg (n=3) | **Intermediate Dose - Single Prime** 2.5x10^6^ live cells/kg (n=5) | **Intermediate Dose - Double Prime** 2.5x10^6^ live cells/kg DP (n=4) | **High Dose –**  **Double Prime** 5x10^6^ live cells/kg DP (n=6) |  | **Total (N=18)** | |
| --- | --- | --- | --- | --- | --- | --- | --- | --- |
| Any Treatment Emergent AE | | 3 (100.0) | 5 (100.0) | 4 (100.0) | 5 (83.3) |  | 17 (94.4) | |
|  |  | | | | | | |  |
| Fatigue | | 0 | 2 (40.0) | 0 | 4 (66.7) |  | 6 (33.3) | |
| Dizziness | | 1 (33.3) | 1 (20.0) | 1 (25.0) | 1 (16.7) |  | 4 (22.2) | |
| Dyspnoea | | 0 | 2 (40.0) | 1 (25.0) | 1 (16.7) |  | 4 (22.2) | |
| Dehydration | | 1 (33.3) | 1 (20.0) | 1 (25.0) | 0 |  | 3 (16.7) | |
| Diarrhoea | | 0 | 2 (40.0) | 1 (25.0) | 0 |  | 3 (16.7) | |
| Flushing | | 1 (33.3) | 0 | 0 | 2 (33.3) |  | 3 (16.7) | |
| Hypotension | | 2 (66.7) | 1 (20.0) | 0 | 0 |  | 3 (16.7) | |
| Nausea | | 0 | 1 (20.0) | 0 | 2 (33.3) |  | 3 (16.7) | |
| Oedema peripheral | | 0 | 0 | 2 (50.0) | 1 (16.7) |  | 3 (16.7) | |
| Urinary tract infection | | 0 | 2 (40.0) | 1 (25.0) | 0 |  | 3 (16.7) | |
| Weight decreased | | 0 | 1 (20.0) | 1 (25.0) | 1 (16.7) |  | 3 (16.7) | |
| Abdominal distension | | 1 (33.3) | 0 | 1 (25.0) | 0 |  | 2 (11.1) | |
| Anaemia | | 0 | 1 (20.0) | 1 (25.0) | 0 |  | 2 (11.1) | |
| Anxiety | | 1 (33.3) | 1 (20.0) | 0 | 0 |  | 2 (11.1) | |
| Constipation | | 0 | 0 | 1 (25.0) | 1 (16.7) |  | 2 (11.1) | |
| Cough | | 1 (33.3) | 0 | 0 | 1 (16.7) |  | 2 (11.1) | |
| Depression | | 1 (33.3) | 0 | 1 (25.0) | 0 |  | 2 (11.1) | |
| Ear pain | | 0 | 1 (20.0) | 0 | 1 (16.7) |  | 2 (11.1) | |
| Hyperuricaemia | | 0 | 0 | 1 (25.0) | 1 (16.7) |  | 2 (11.1) | |
| Hypomagnesaemia | | 1 (33.3) | 0 | 1 (25.0) | 0 |  | 2 (11.1) | |
| Hyponatraemia | | 0 | 0 | 2 (50.0) | 0 |  | 2 (11.1) | |
| Infusion related reaction | | 1 (33.3) | 0 | 1 (25.0) | 0 |  | 2 (11.1) | |
| Myalgia | | 0 | 0 | 1 (25.0) | 1 (16.7) |  | 2 (11.1) | |
| Non-cardiac chest pain | | 0 | 0 | 2 (50.0) | 0 |  | 2 (11.1) | |
| Oropharyngeal pain | | 0 | 1 (20.0) | 0 | 1 (16.7) |  | 2 (11.1) | |
| Pleural effusion | | 0 | 0 | 2 (50.0) | 0 |  | 2 (11.1) | |
| Procedural pain | | 0 | 0 | 2 (50.0) | 0 |  | 2 (11.1) | |
| Pruritus | | 0 | 1 (20.0) | 0 | 1 (16.7) |  | 2 (11.1) | |
| Urinary retention | | 1 (33.3) | 0 | 1 (25.0) | 0 |  | 2 (11.1) | |
| Wheezing | | 1 (33.3) | 0 | 0 | 1 (16.7) |  | 2 (11.1) | |
| Activated partial thromboplastin time prolonged | | 0 | 0 | 0 | 1 (16.7) |  | 1 (5.6) | |
| Acute respiratory failure | | 0 | 0 | 1 (25.0) | 0 |  | 1 (5.6) | |
| Bile duct obstruction | | 0 | 1 (20.0) | 0 | 0 |  | 1 (5.6) | |
| Blood alkaline phosphatase increased | | 0 | 0 | 0 | 1 (16.7) |  | 1 (5.6) | |
| Blood bilirubin increased | | 0 | 0 | 1 (25.0) | 0 |  | 1 (5.6) | |
| Blood creatinine increased | | 0 | 0 | 0 | 1 (16.7) |  | 1 (5.6) | |
| Chills | | 1 (33.3) | 0 | 0 | 0 |  | 1 (5.6) | |

**Table 2 cont.**

| **MedDRA**  **Preferred Term** | **Low Dose -**  **Single Prime** 0.5x10^6^ live cells/kg (n=3) | **Intermediate Dose - Single Prime** 2.5x10^6^ live cells/kg (n=5) | **Intermediate Dose - Double Prime** 2.5x10^6^ live cells/kg DP (n=4) | **High Dose –**  **Double Prime** 5x10^6^ live cells/kg DP (n=6) | **Total (N=18)** |
| --- | --- | --- | --- | --- | --- |
| Chronic obstructive pulmonary disease | 0 | 1 (20.0) | 0 | 0 | 1 (5.6) |
| Corona virus infection | 0 | 0 | 1 (25.0) | 0 | 1 (5.6) |
| Cytokine release syndrome | 1 (33.3) | 0 | 0 | 0 | 1 (5.6) |
| Deafness | 0 | 0 | 0 | 1 (16.7) | 1 (5.6) |
| Dyspepsia | 1 (33.3) | 0 | 0 | 0 | 1 (5.6) |
| Dysphagia | 0 | 0 | 0 | 1 (16.7) | 1 (5.6) |
| Fungal infection | 0 | 0 | 1 (25.0) | 0 | 1 (5.6) |
| Headache | 0 | 1 (20.0) | 0 | 0 | 1 (5.6) |
| Hyperkalaemia | 1 (33.3) | 0 | 0 | 0 | 1 (5.6) |
| Hyperphosphataemia | 1 (33.3) | 0 | 0 | 0 | 1 (5.6) |
| Hypoalbuminaemia | 0 | 0 | 1 (25.0) | 0 | 1 (5.6) |
| Hypokalaemia | 0 | 1 (20.0) | 0 | 0 | 1 (5.6) |
| Immunisation anxiety related reaction | 0 | 0 | 0 | 1 (16.7) | 1 (5.6) |
| Insomnia | 0 | 0 | 1 (25.0) | 0 | 1 (5.6) |
| Joint range of motion decreased | 1 (33.3) | 0 | 0 | 0 | 1 (5.6) |
| Lymph node pain | 1 (33.3) | 0 | 0 | 0 | 1 (5.6) |
| Lymphopenia | 0 | 0 | 0 | 1 (16.7) | 1 (5.6) |
| Malaise | 1 (33.3) | 0 | 0 | 0 | 1 (5.6) |
| Malignant neoplasm progression | 0 | 0 | 1 (25.0) | 0 | 1 (5.6) |
| Muscular weakness | 1 (33.3) | 0 | 0 | 0 | 1 (5.6) |
| Myoclonus | 1 (33.3) | 0 | 0 | 0 | 1 (5.6) |
| Nasal congestion | 1 (33.3) | 0 | 0 | 0 | 1 (5.6) |
| Neck injury | 0 | 0 | 0 | 1 (16.7) | 1 (5.6) |
| Neuropathy peripheral | 0 | 0 | 0 | 1 (16.7) | 1 (5.6) |
| Oral herpes | 1 (33.3) | 0 | 0 | 0 | 1 (5.6) |
| Pain in extremity | 1 (33.3) | 0 | 0 | 0 | 1 (5.6) |
| Pancytopenia | 0 | 0 | 0 | 1 (16.7) | 1 (5.6) |
| Pericardial effusion | 0 | 0 | 1 (25.0) | 0 | 1 (5.6) |
| Perineal infection | 1 (33.3) | 0 | 0 | 0 | 1 (5.6) |
| Presyncope | 1 (33.3) | 0 | 0 | 0 | 1 (5.6) |
| Rash pruritic | 0 | 0 | 0 | 1 (16.7) | 1 (5.6) |
| Rectal haemorrhage | 1 (33.3) | 0 | 0 | 0 | 1 (5.6) |
| Sepsis | 0 | 1 (20.0) | 0 | 0 | 1 (5.6) |
| Somnolence | 0 | 0 | 1 (25.0) | 0 | 1 (5.6) |
| Swelling | 0 | 0 | 0 | 1 (16.7) | 1 (5.6) |
| Tachypnoea | 0 | 0 | 1 (25.0) | 0 | 1 (5.6) |
| Tumour pain | 0 | 0 | 0 | 1 (16.7) | 1 (5.6) |
| Urinary incontinence | 1 (33.3) | 0 | 0 | 0 | 1 (5.6) |
| Vomiting | 0 | 1 (20.0) | 0 | 0 | 1 (5.6) |
| Vulvitis | 1 (33.3) | 0 | 0 | 0 | 1 (5.6) |

**Table S3.** All related TEAEs from escalating dose evaluation of SQZ-PBMC-HPV, with breakout by grade. Entries give the number of subjects and the corresponding percentage of the number of patients in the respective cohort, or the study in its entirety.

| **MedDRA**  **Preferred Term** | **Low Dose - Single Prime** 0.5x10^6^ live cells/kg (n=3) | **Intermediate Dose - Single Prime** 2.5x10^6^ live cells/kg (n=5) | **Intermediate Dose - Double Prime** 2.5x10^6^ live cells/kg DP (n=4) | **High Dose - Double Prime** 5x10^6^ live cells/kg DP (n=6) | **Total (N=18)** |
| --- | --- | --- | --- | --- | --- |
| **Any Treatment Emergent AE** | **3 (100.0)** | **4 (80.0)** | **2 (50.0)** | **5 (83.3)** | **14 (77.8)** |
|  | | | | | |
| **Fatigue** | **0** | **1 (20.0)** | **0** | **4 (66.7)** | **5 (27.8)** |
| Grade 1 | 0 | 1 (20.0) | 0 | 3 (50.0) | 4 (22.2) |
| Grade 2 | 0 | 0 | 0 | 1 (16.7) | 1 (5.6) |
| **Flushing** | **1 (33.3)** | **0** | **0** | **2 (33.3)** | **3 (16.7)** |
| Grade 1 | 1 (33.3) | 0 | 0 | 2 (33.3) | 3 (16.7) |
| **Hypotension** | **2 (66.7)** | **1 (20.0)** | **0** | **0** | **3 (16.7)** |
| Grade 1 | 1 (33.3) | 0 | 0 | 0 | 1 (5.6) |
| Grade 2 | 1 (33.3) | 1 (20.0) | 0 | 0 | 2 (11.1) |
| **Infusion related reaction** | **1 (33.3)** | **0** | **1 (25.0)** | **0** | **2 (11.1)** |
| Grade 1 | 0 | 0 | 1 (25.0) | 0 | 1 (5.6) |
| Grade 2 | 1 (33.3) | 0 | 0 | 0 | 1 (5.6) |
| **Nausea** | **0** | **0** | **0** | **2 (33.3)** | **2 (11.1)** |
| Grade 1 | 0 | 0 | 0 | 1 (16.7) | 1 (5.6) |
| Grade 2 | 0 | 0 | 0 | 1 (16.7) | 1 (5.6) |
| **Pruritus** | **0** | **1 (20.0)** | **0** | **1 (16.7)** | **2 (11.1)** |
| Grade 1 | 0 | 0 | 0 | 1 (16.7) | 1 (5.6) |
| Grade 2 | 0 | 1 (20.0) | 0 | 0 | 1 (5.6) |
| **Anaemia** | **0** | **1 (20.0)** | **0** | **0** | **1 (5.6)** |
| Grade 3 | 0 | 1 (20.0) | 0 | 0 | 1 (5.6) |
| **Blood creatinine increased** | **0** | **0** | **0** | **1 (16.7)** | **1 (5.6)** |
| Grade 1 | 0 | 0 | 0 | 1 (16.7) | 1 (5.6) |
| **Chills** | **1 (33.3)** | **0** | **0** | **0** | **1 (5.6)** |
| Grade 2 | 1 (33.3) | 0 | 0 | 0 | 1 (5.6) |
| **Cough** | **1 (33.3)** | **0** | **0** | **0** | **1 (5.6)** |
| Grade 1 | 1 (33.3) | 0 | 0 | 0 | 1 (5.6) |
| **Cytokine release syndrome** | **1 (33.3)** | **0** | **0** | **0** | **1 (5.6)** |
| Grade 2 | 1 (33.3) | 0 | 0 | 0 | 1 (5.6) |
| **Dyspnoea** | **0** | **1 (20.0)** | **0** | **0** | **1 (5.6)** |
| Grade 1 | 0 | 1 (20.0) | 0 | 0 | 1 (5.6) |
| **Hyponatraemia** | **0** | **0** | **1 (25.0)** | **0** | **1 (5.6)** |
| Grade 2 | 0 | 0 | 1 (25.0) | 0 | 1 (5.6) |

**Table S3 cont.**

| **MedDRA**  **Preferred Term** | **Low Dose - Single Prime** 0.5x10^6^ live cells/kg (n=3) | **Intermediate Dose - Single Prime** 2.5x10^6^ live cells/kg (n=5) | **Intermediate Dose - Double Prime** 2.5x10^6^ live cells/kg DP (n=4) | **High Dose - Double Prime** 5x10^6^ live cells/kg DP (n=6) | **Total (N=18)** |
| --- | --- | --- | --- | --- | --- |
| **Any Treatment Emergent AE** | **3 (100.0)** | **4 (80.0)** | **2 (50.0)** | **5 (83.3)** | **14 (77.8)** |
|  | | | | | |
| **Immunisation anxiety related reaction** | **0** | **0** | **0** | **1 (16.7)** | **1 (5.6)** |
| Grade 1 | 0 | 0 | 0 | 1 (16.7) | 1 (5.6) |
| **Lymph node pain** | **1 (33.3)** | **0** | **0** | **0** | **1 (5.6)** |
| Grade 1 | 1 (33.3) | 0 | 0 | 0 | 1 (5.6) |
| **Malaise** | **1 (33.3)** | **0** | **0** | **0** | **1 (5.6)** |
| Grade 1 | 1 (33.3) | 0 | 0 | 0 | 1 (5.6) |
| **Myalgia** | **0** | **0** | **0** | **1 (16.7)** | **1 (5.6)** |
| Grade 1 | 0 | 0 | 0 | 1 (16.7) | 1 (5.6) |
| **Nasal congestion** | **1 (33.3)** | **0** | **0** | **0** | **1 (5.6)** |
| Grade 2 | 1 (33.3) | 0 | 0 | 0 | 1 (5.6) |
| **Pancytopenia** | **0** | **0** | **0** | **1 (16.7)** | **1 (5.6)** |
| Grade 2 | 0 | 0 | 0 | 1 (16.7) | 1 (5.6) |
| **Rash pruritic** | **0** | **0** | **0** | **1 (16.7)** | **1 (5.6)** |
| Grade 1 | 0 | 0 | 0 | 1 (16.7) | 1 (5.6) |
| **Weight decreased** | **0** | **0** | **0** | **1 (16.7)** | **1 (5.6)** |
| Grade 2 | 0 | 0 | 0 | 1 (16.7) | 1 (5.6) |
| **Wheezing** | **1 (33.3)** | **0** | **0** | **0** | **1 (5.6)** |
| Grade 2 | 1 (33.3) | 0 | 0 | 0 | 1 (5.6) |

**Table S4.** Summary of best overall response per RECIST1.1 and percent change in CD8+ tumor infiltrating lymphocytes, E6 transcripts, and E7 transcripts as determined by ISH comparing baseline biopsy and cycle 2, day 8 biopsy.

PD: Progressive Disease; SD: Stable Disease; PR: Partial Response

| **Cohort** | **Patient ID** | **Best Overall Response** | **CD8+ % Change from Baseline** | **E6 % Change from Baseline** | **E7 % Change from Baseline** |
| --- | --- | --- | --- | --- | --- |
| **Low Dose - Single Prime** 0.5x10^6^ live cells/kg | 1 | SD | -66.1% | -17.5% | -17.7% |
|  | 2 | SD | 101.5% | 9.3% | 22.2% |
|  | 3 | SD | 8.6% | 93.6% | -12.6% |
| **Intermediate Dose - Single Prime** 2.5x10^6^ live cells/kg | 4 | PD | -36.4% | --- | --- |
|  | 5 | PD | -78.4% | --- | --- |
|  | 6 | PD | --- | --- | --- |
|  | 7 | SD | 542.6% | --- | --- |
|  | 8 | PD | --- | --- | --- |
| **Intermediate Dose - Double Prime** 2.5x10^6^ live cells/kg | 9 | PD | 17.9% | --- | --- |
|  | 10 | PD | -37.1% | --- | --- |
|  | 11 | --- | 22.0% | --- | --- |
|  | 12 | PD | -78.2% | --- | --- |
| **High Dose - Double Prime** 5x10^6^ live cells/kg | 13 | PD | -24.2% | --- | --- |
|  | 14 | PD | -15.7% | -9.9% | 25.8% |
|  | 15 | PD | -40.8% | -27.2% | -16.9% |
|  | 16 | PD | -63.8% | -28.7% | -27.0% |
|  | 17 | PR | 712.3% | -91.1% | -91.5% |
|  | 18 | --- | -27.1% | -12.8% | -11.2% |

**CONSORT**

High Dose - Double Prime

5.0x10^6^ live cells/kg DP (n = 8)

- Received treatment (n=6)
- Did not receive treatment (n=2)
  - Pending C1D1 (n=1)
  - PD before C1D1 (n=1)

Discontinued Treatment (n=4)

- Progressive Disease (n=2)
- Completed Tx/Exhausted Product (n=2)

Low Dose - Single Prime

0.5x10^6^ live cells/kg (n = 3)

- Received treatment (n=3)
- Did not receive treatment (n=0)

Intermediate Dose - Double Prime

2.5x10^6^ live cells/kg DP (n = 4)

- Received treatment (n=4)
- Did not receive treatment (n=0)

Intermediate Dose - Single Prime

2.5x10^6^ live cells/kg (n = 5)

- Received treatment (n=5)
- Did not receive treatment (n=0)

Discontinued Treatment (n=3)

- Progressive Disease (n=1)
- Completed Tx/Exhausted Product (n=2)

Discontinued Treatment (n=5)

- Progressive Disease (n=3)
- Completed Tx/Exhausted Product (n=1)
- Withdrew Consent (n=1)

Discont. Treatment (n=4)

- Progressive Disease (n=3)
- Death (n=1)

Off Study (n=1)

- Death (n=1)

Off Study (n=4)

- Death (n=4)

Off Study (n=3)

- Death (n=3)

Consented (n=84)

Enrolled (n=20)

Not Enrolled (n=64)

- Not HPV16+ (n=7)
- Not HLA*A02+ (n=35)
- Not HPV16+/HLA*A02+ (n=3)
- Other (n=19)

Off Study (n=0)

Allocation, Leukapheresis, and Treatment

Follow Up

Follow Up

Off Study

Off Study

**Figure S1.** Consort flow diagram showing stages in screening, enrollment, treatment, and follow-up for participants for the Phase 1 monotherapy assessment of SQZ-PBMC-HPV.


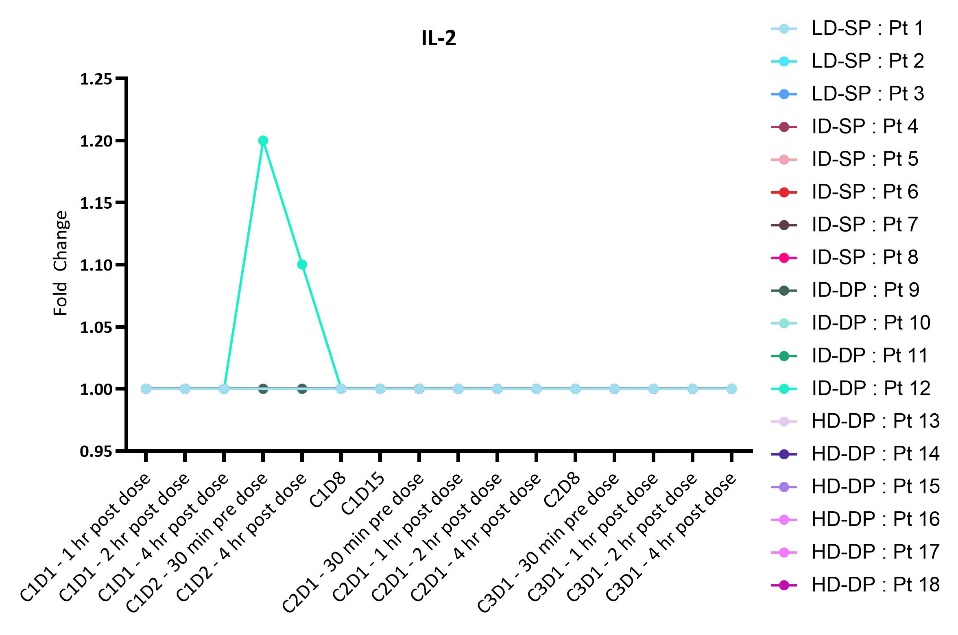


**A**


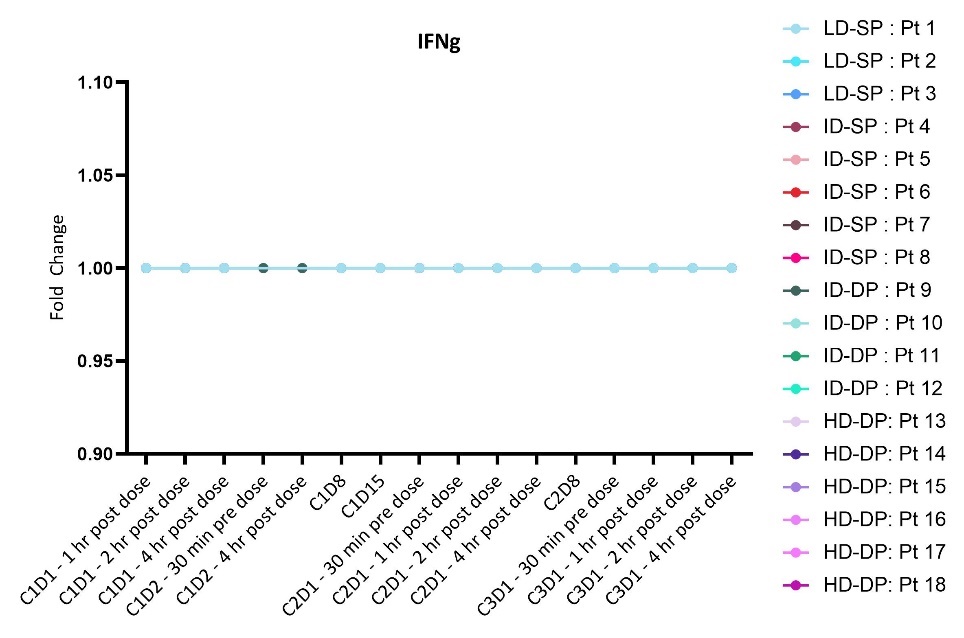


**B**


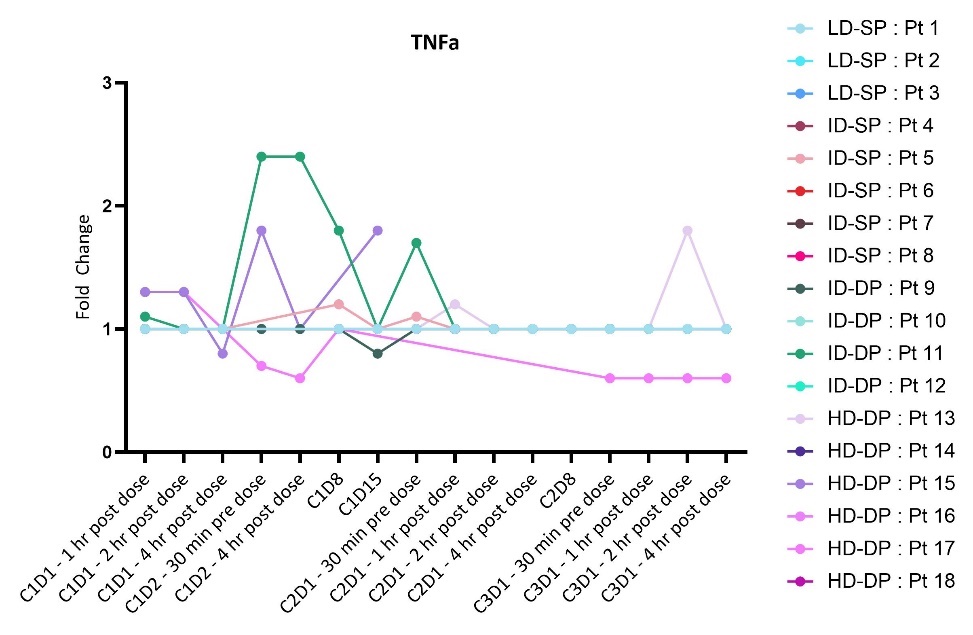


**C**

**Figure S2.** SQZ-PBMV-HPV is not associated with systemic inflammation. Serum cytokine levels for subjects receiving SQZ-PBMC-HPV. Graph reports relative fold changes of indicated sytokine, compared to the baseline sample drawn 30 minutes before cycle 1 day 1 administration. Baseline samples were all within accepted range of normal. X-axis is by patient visit and is not linear. Y-axes have different scales. **A:** IL-2 levels; **B:** IFNɣ levels; **C:** TNFɑ levels.

Note: all patients received scheduled doses of SQZ-PBMC-HPV for cycles 1 and 2, then differed in duration of treatment thereafter. Cohort Key: LD-SP: Low Dose – Single Prime; ID-SP: Intermediate Dose – Single Prime; ID-DP: Intermediate Dose – Double Prime; HD-DP: High Dose– Double Prime.


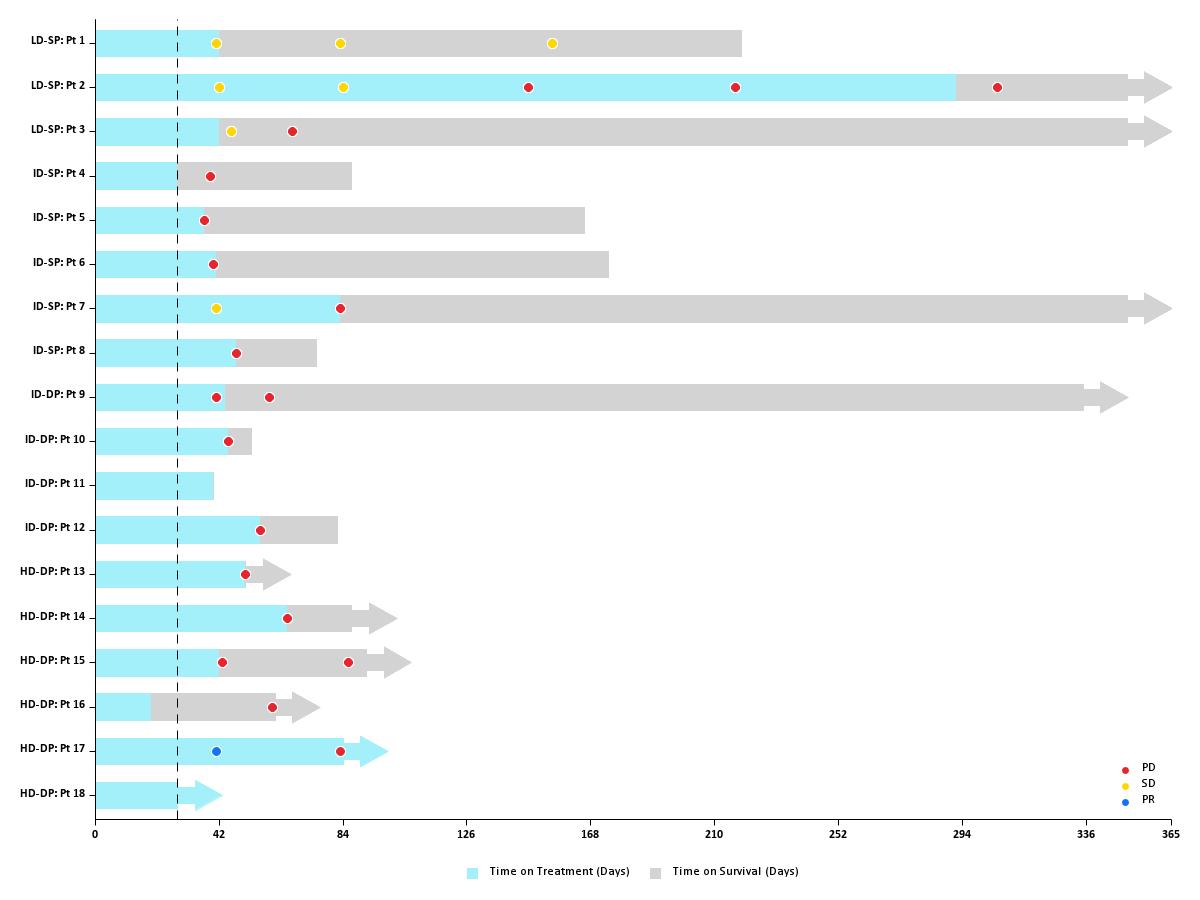


**Figure S3.** Swimmer plot showing time on SQZ-PBMC-HPV treatment and overall survival up to one year and tumor response per RECIST1.1. The end of the dose limiting toxicity (DLT) period is shown as a dashed line. All patients were DLT evaluable.

PD: Progressive Disease; SD: Stable Disease; PR: Partial Response


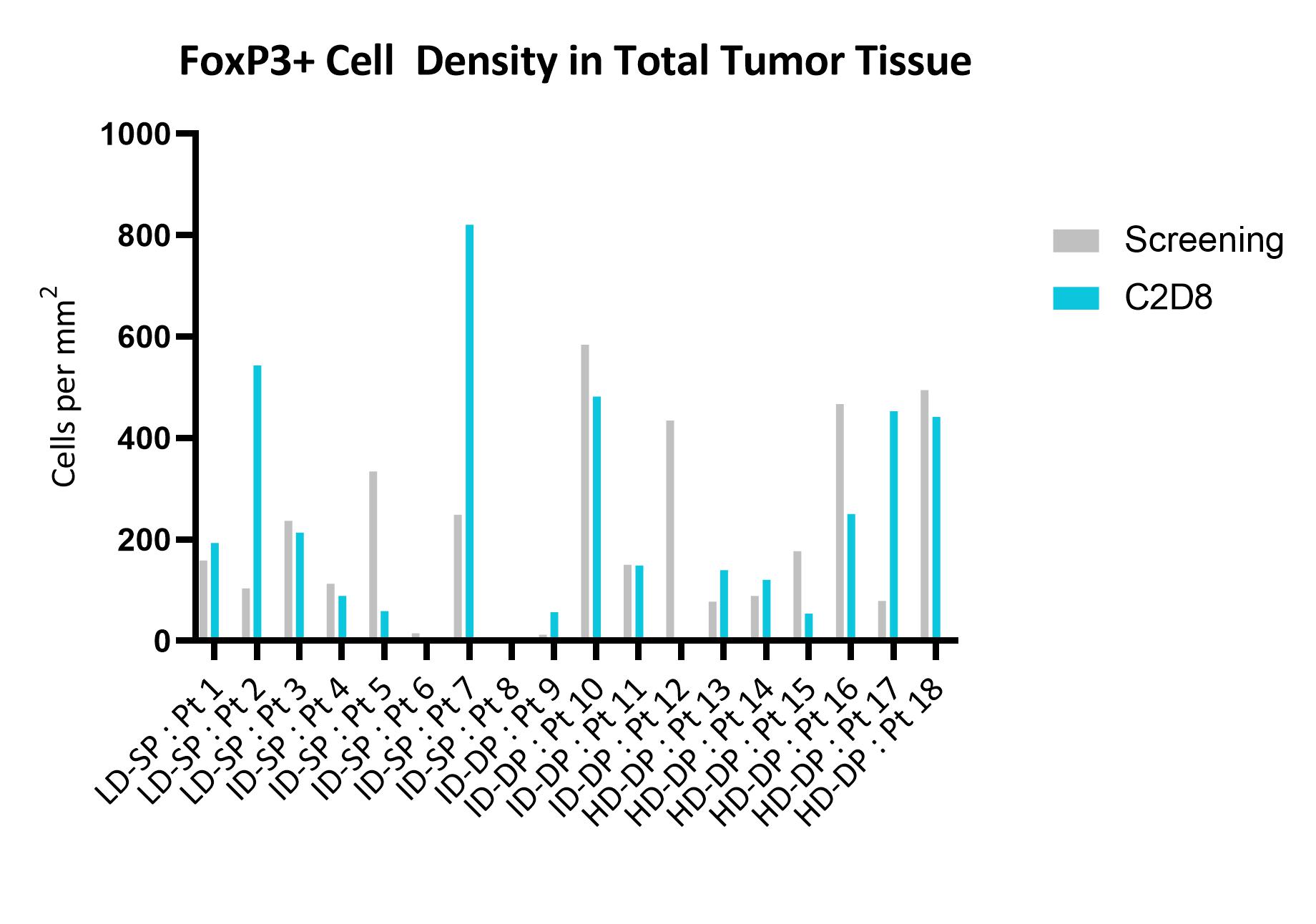


**Figure S4.** Histology results for FoxP3+ cell density in tumor tissue, utilizing paired biopsies from subjects in escalating dose evaluation of SQZ-PBMC-HPV. The pretreatment biopsy was obtained within 28 days of the subject’s leukapheresis, and the comparative sample obtained on cycle 2, day 8, at the conclusion of the DLT observation period. At this point, subjects in the single prime cohorts had received 2 infusions of their autologous SQZ-PBMC-HPV; subjects on the double prime cohort will have received three infusions.


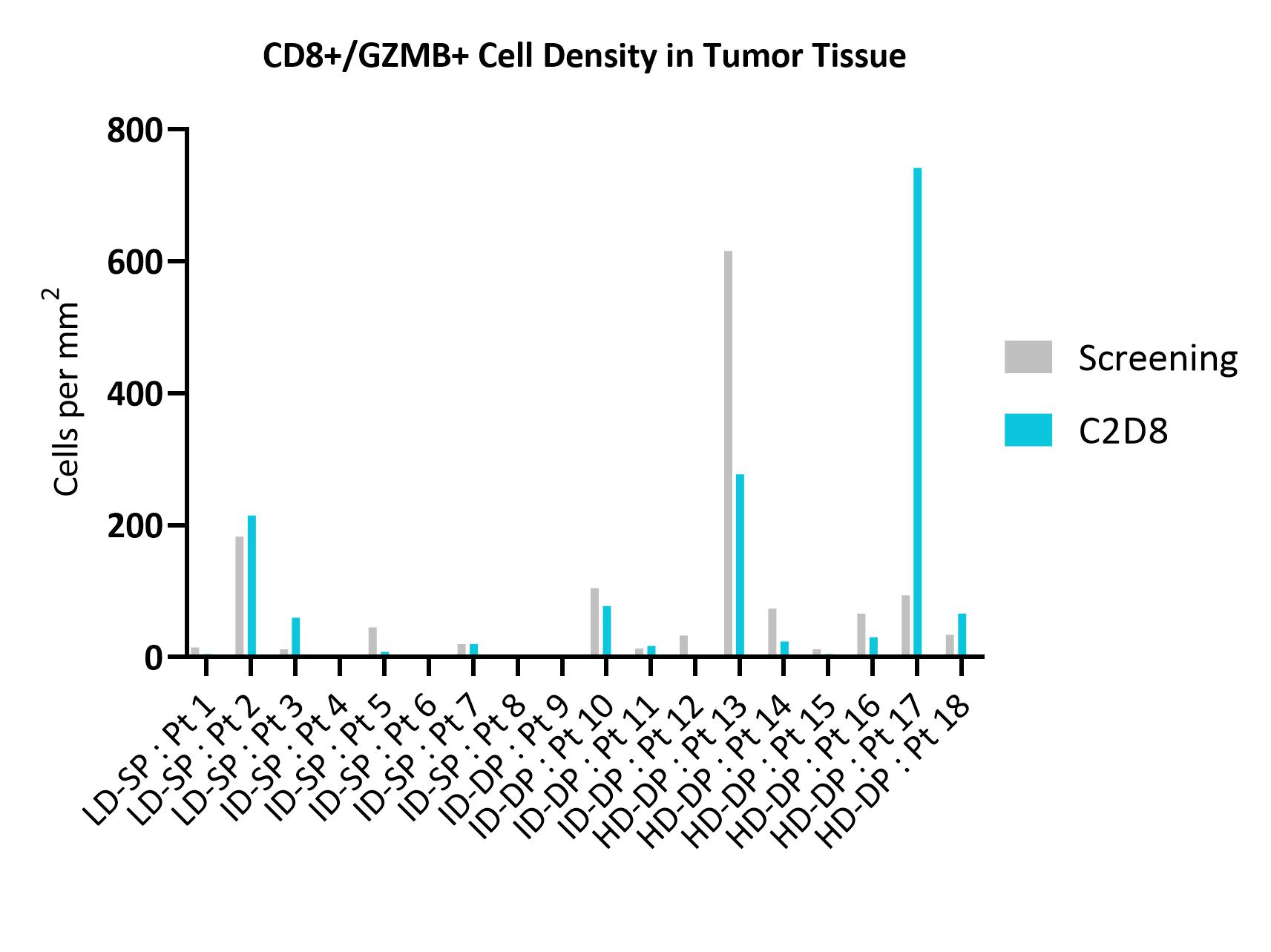


**Figure S5.** Histology results for CD8+/GZMB+ cell density in tumor tissue, utilizing paired biopsies from subjects in escalation dose evaluation of SQZ-PBMC-HPV. The pretreatment biopsy was obtained within 28 days of the subject’s leukapheresis, and the comparative sample obtained on cycle 2, day 8, at the conclusion of the DLT observation period. At this point, subjects in the single prime cohorts had received 2 infusions of their autologous SQZ-PBMC-HPV; subjects in the double prime cohort will have received three infusions.


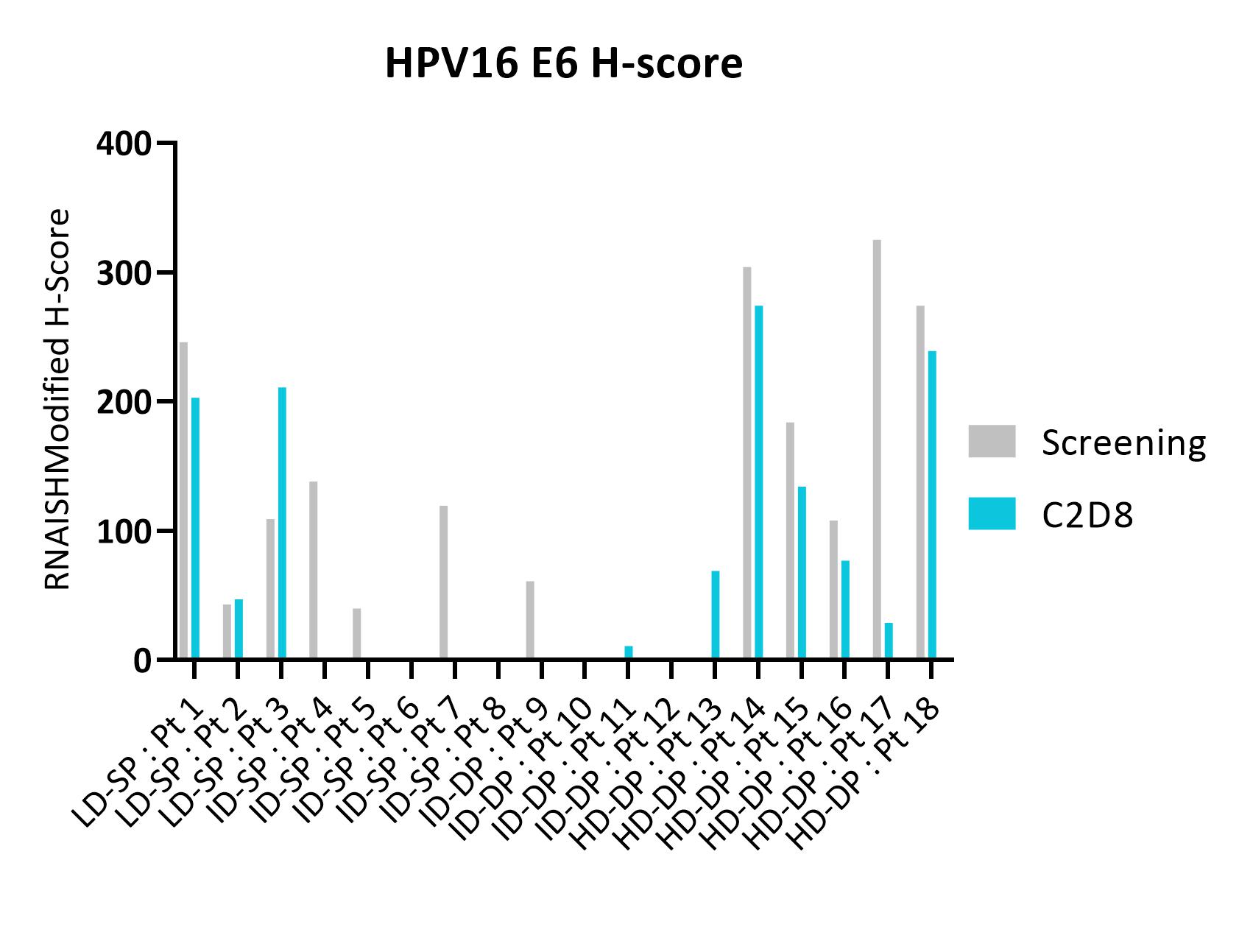


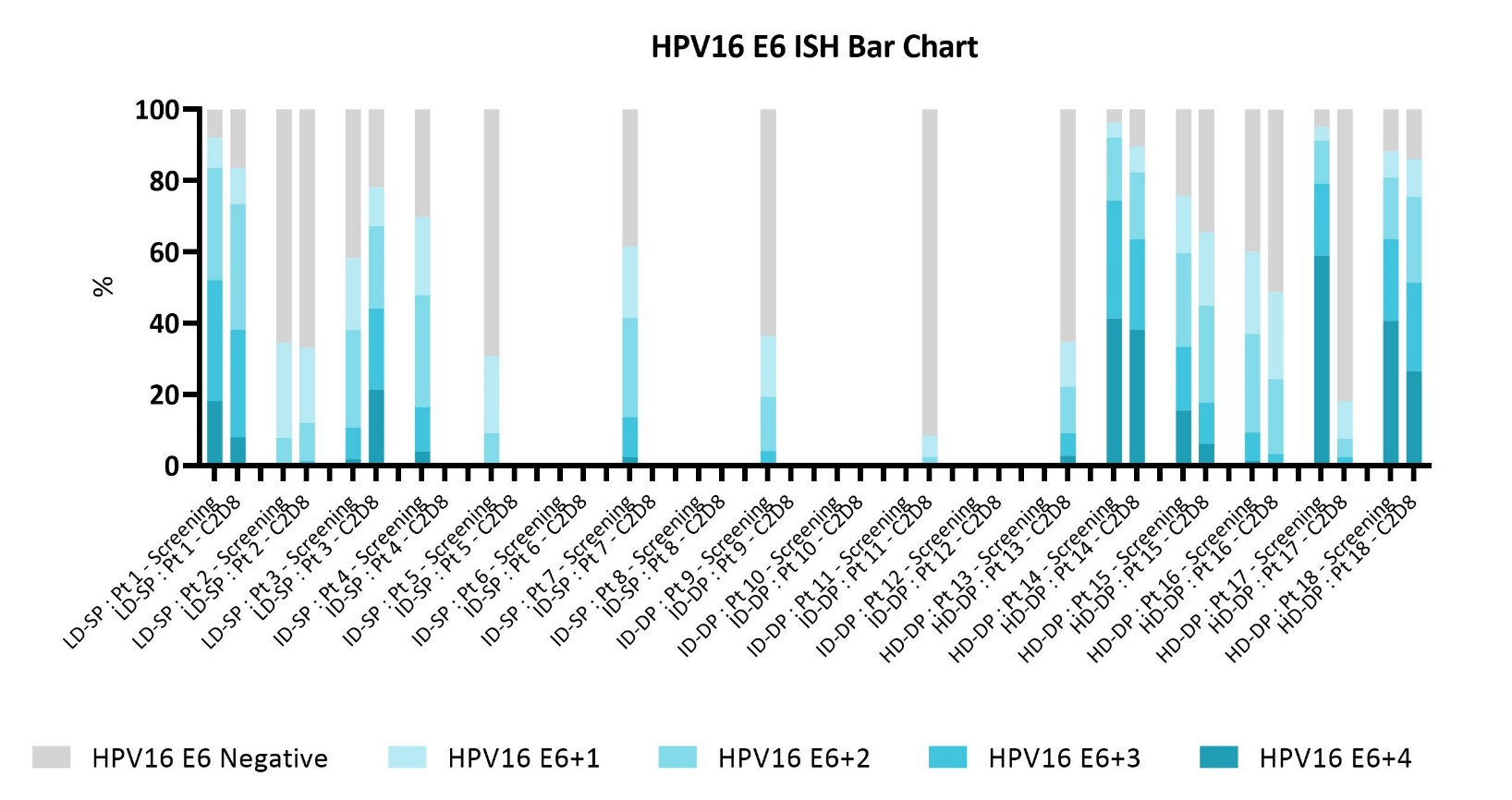


**Figure S6.** Transcript levels of HPV16 E6 in paired biopsies from subjects in escalating dose evaluation of SQZ-PBMC-HPV. Top panel shows aggregate level of transcript as the RNA ISH Modified H-Score. Lower panel shows semi-quantitative binning of ISH intensity for each biopsy. The pretreatment biopsy was obtained within 28 days of the subject’s leukapheresis, and the comparative sample obtained on cycle 2, day 8, at the conclusion of the DLT observation period.


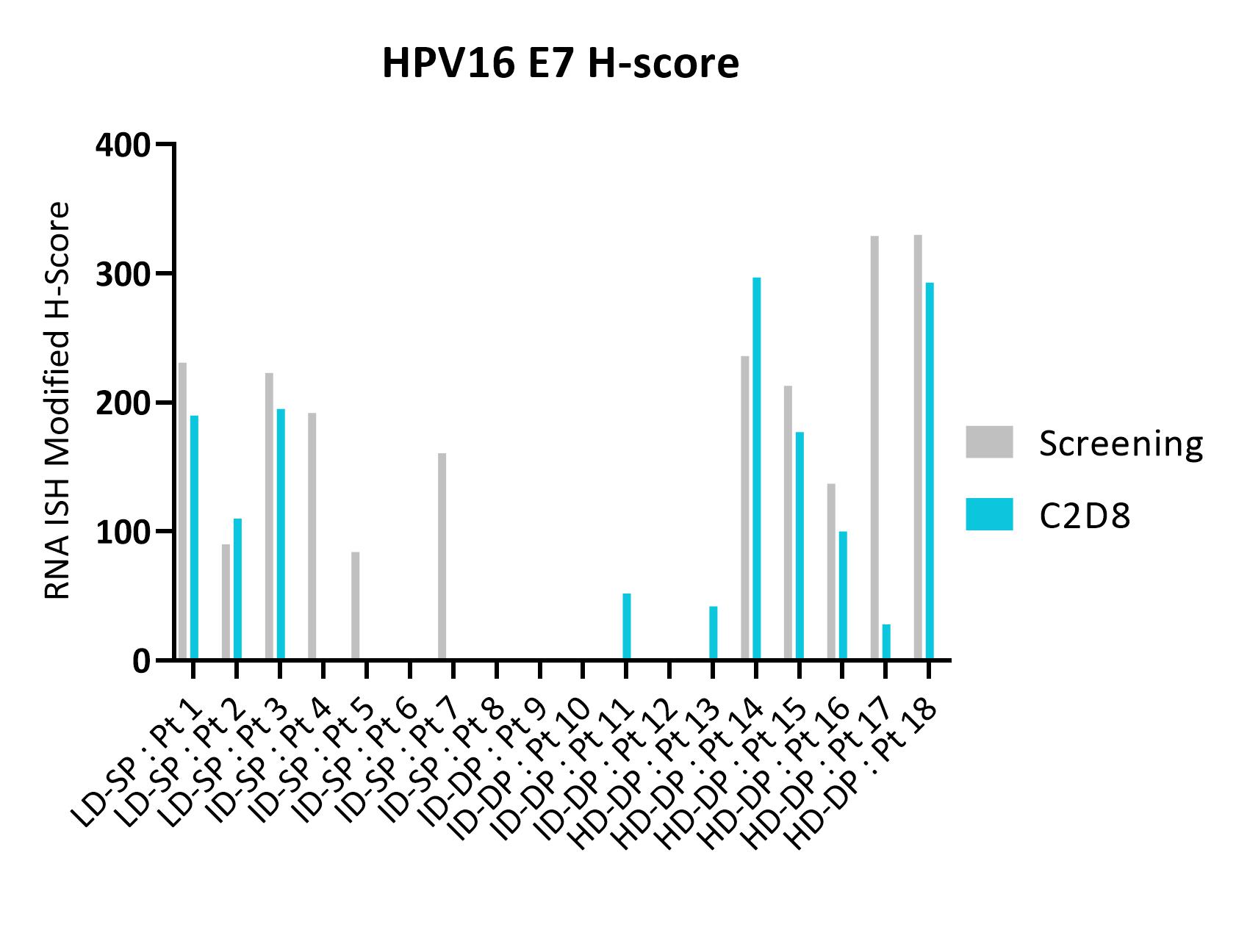


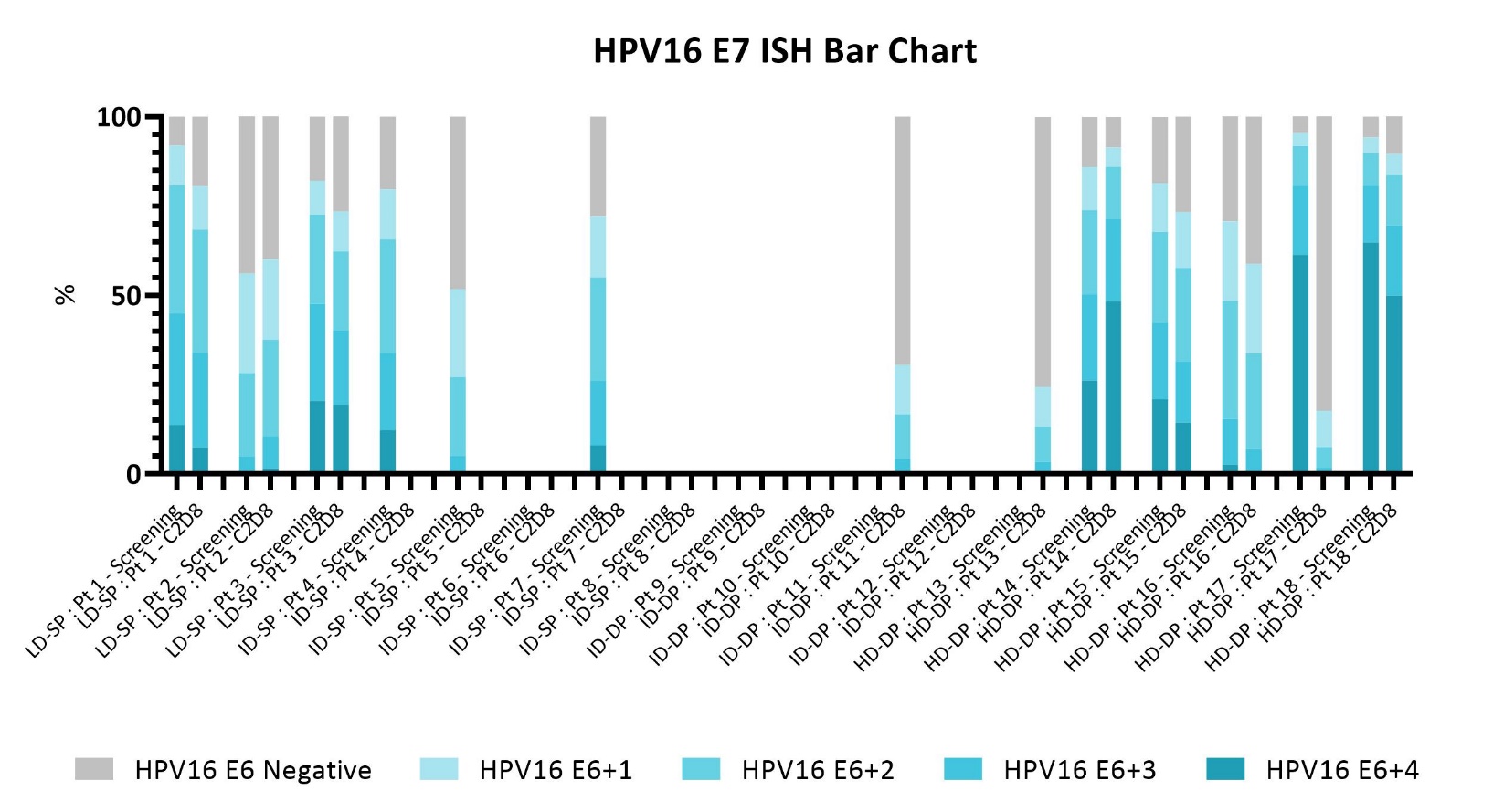


**Figure S7.** Transcript levels of HPV16 E7 by *in situ* hybridization, in paired biopsies from subjects in escalating dose evaluation of SQZ-PBMC-HPV. Top panel shows aggregate level of transcript as the RNA ISH Modified H-Score. Lower panel shows semi-quantitative binning of ISH intensity for each biopsy. The pretreatment biopsy was obtained within 28 days of the subject’s leukapheresis, and the comparative sample obtained on cycle 2, day 8, at the conclusion of the DLT observation period.


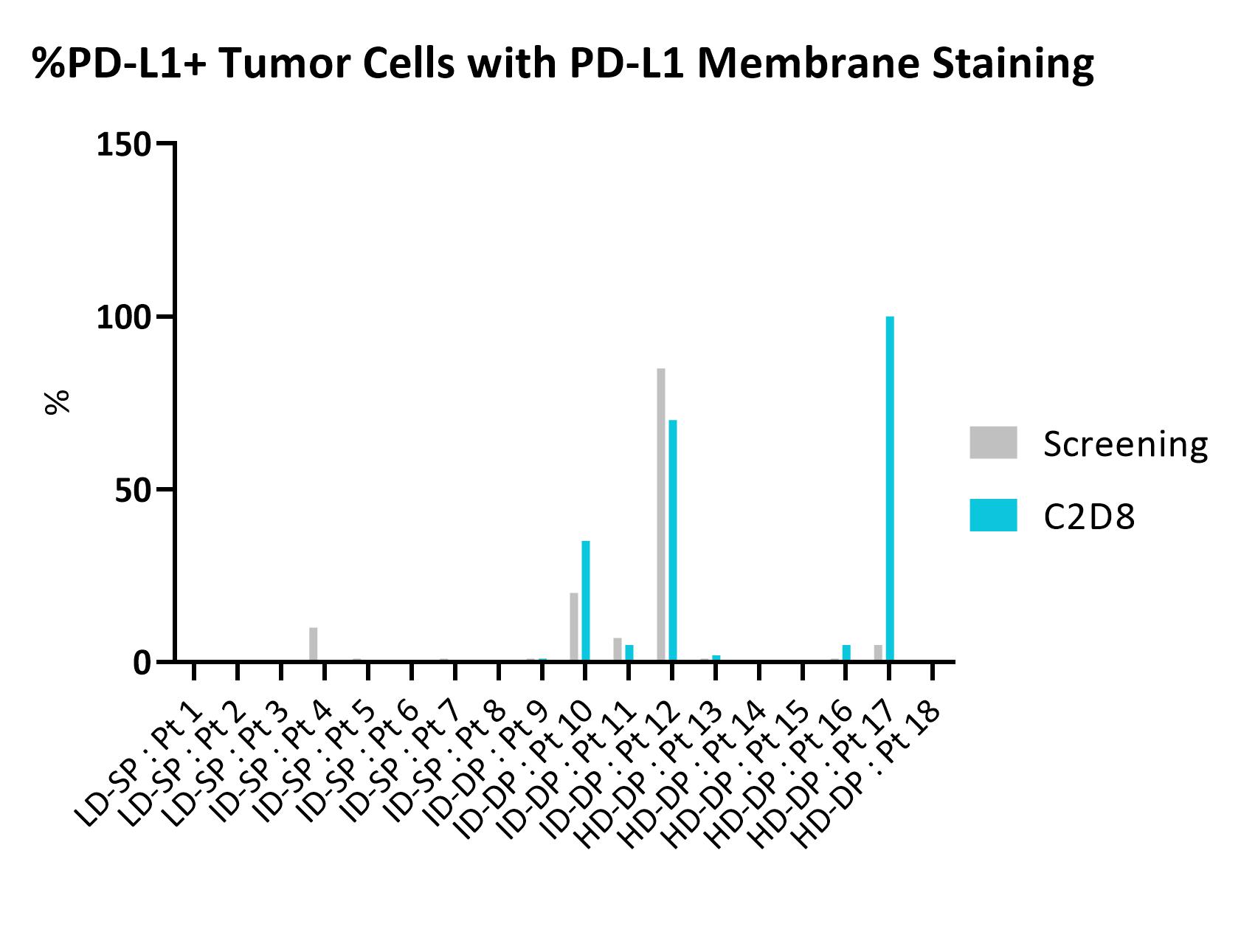


**Figure S8.** Percentage of tumor cells with PD-L1 membrane staining, measured histologically in paired biopsies from subjects in escalating dose evaluation of SQZ-PBMC-HPV. The pretreatment biopsy was obtained within 28 days of the subject’s leukapheresis, and the comparative sample obtained on cycle 2, day 8, at the conclusion of the DLT observation period.


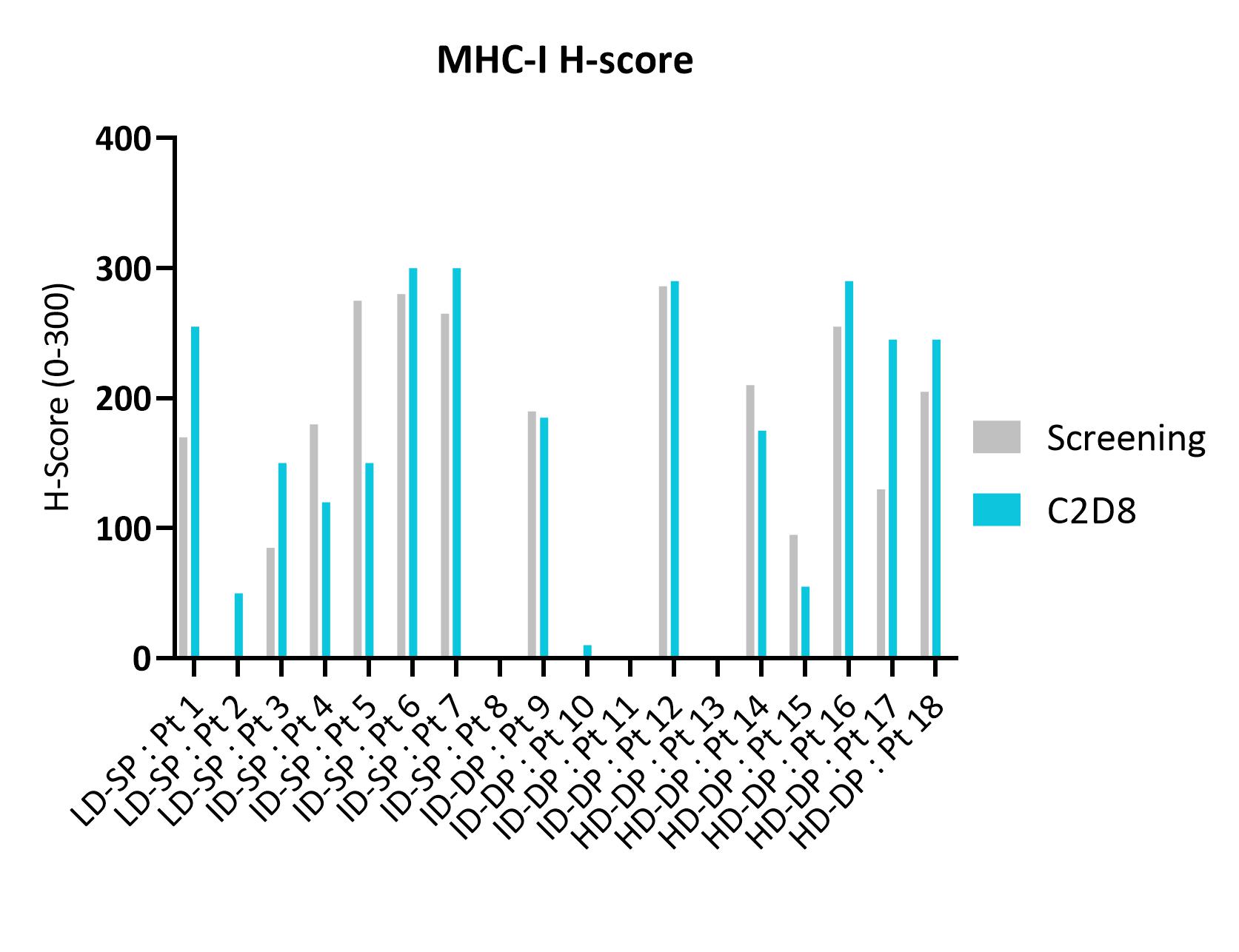


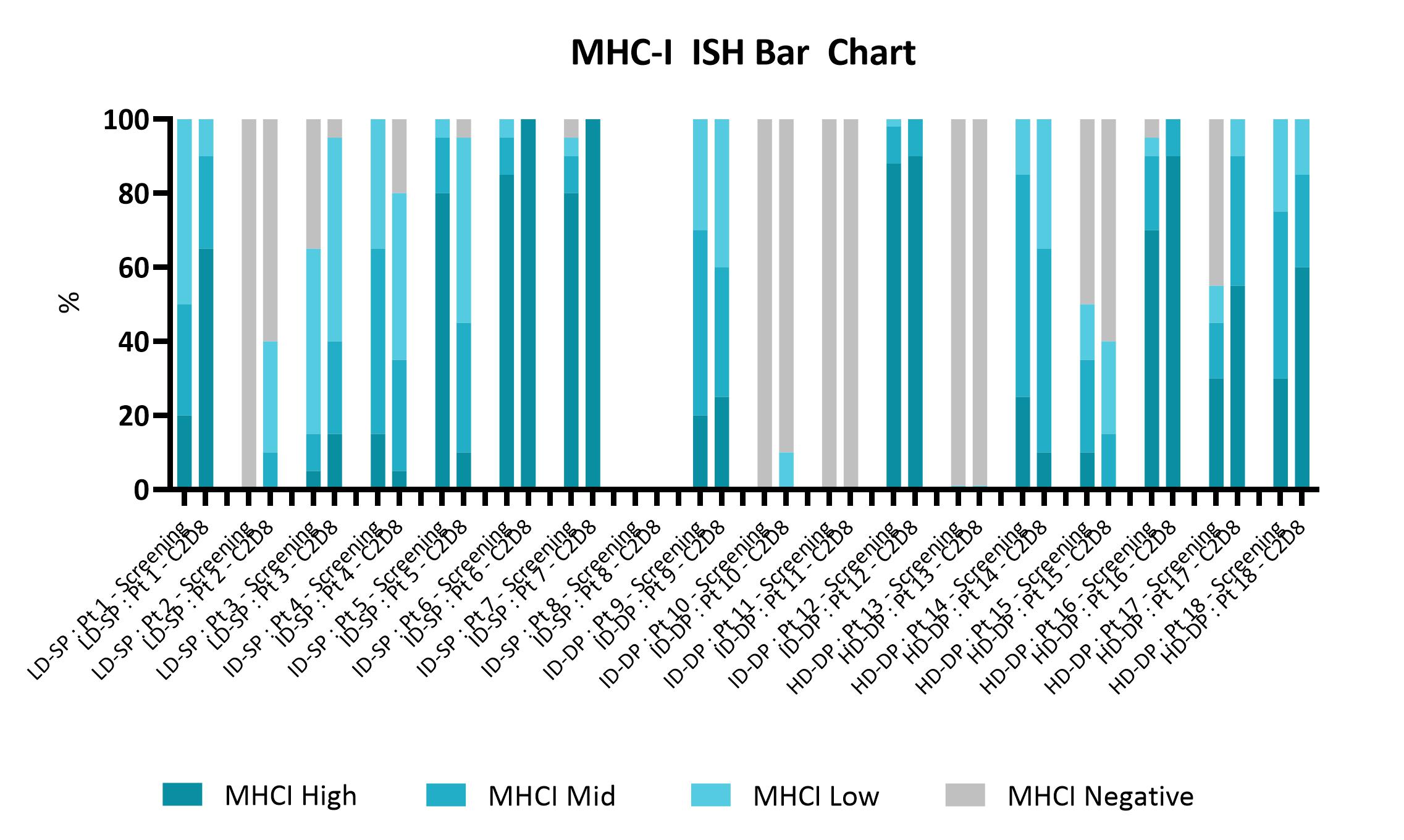


**Figure S9.** MHC-I expression in paired biopsies from subjects in escalating dose evaluation of SQZ-PBMC-HPV. Upper panel graphically plots the H-Score for each paired biopsy. Lower panel furnishes semi-quantitative binning of MHC-I staining, as percentage of cells. The pretreatment biopsy was obtained within 28 days of the subject’s leukapheresis, and the comparative sample obtained on cycle 2, day 8, at the conclusion of the DLT observation period.
